# 3D Recognition Based on Sensor Modalities for Robotic Systems: A Survey

**DOI:** 10.3390/s21217120

**Published:** 2021-10-27

**Authors:** Sumaira Manzoor, Sung-Hyeon Joo, Eun-Jin Kim, Sang-Hyeon Bae, Gun-Gyo In, Jeong-Won Pyo, Tae-Yong Kuc

**Affiliations:** Department of Electrical and Computer Engineering, College of Information and Communication Engineering, Sungkyunkwan University, Suwon 16419, Korea; sumaira11@skku.edu (S.M.); sh.joo@skku.edu (S.-H.J.); eunjin.kim@skku.edu (E.-J.K.); shbae.skku@skku.edu (S.-H.B.); ingungyo@skku.edu (G.-G.I.); jungwon900@skku.edu (J.-W.P.)

**Keywords:** 3D visual recognition, sensors, object detection, place recognition, camera, LiDAR, sensor fusion, deep learning, 3D detection dataset, autonomous vehicles, robotic systems

## Abstract

3D visual recognition is a prerequisite for most autonomous robotic systems operating in the real world. It empowers robots to perform a variety of tasks, such as tracking, understanding the environment, and human–robot interaction. Autonomous robots equipped with 3D recognition capability can better perform their social roles through supportive task assistance in professional jobs and effective domestic services. For active assistance, social robots must recognize their surroundings, including objects and places to perform the task more efficiently. This article first highlights the value-centric role of social robots in society by presenting recently developed robots and describes their main features. Instigated by the recognition capability of social robots, we present the analysis of data representation methods based on sensor modalities for 3D object and place recognition using deep learning models. In this direction, we delineate the research gaps that need to be addressed, summarize 3D recognition datasets, and present performance comparisons. Finally, a discussion of future research directions concludes the article. This survey is intended to show how recent developments in 3D visual recognition based on sensor modalities using deep-learning-based approaches can lay the groundwork to inspire further research and serves as a guide to those who are interested in vision-based robotics applications.

## 1. Introduction

Today, robotic systems with social characteristics are considered an important keystone in household chores, healthcare services, and modern industrial production [1]. 3D visual recognition is the fundamental component of these social robots. Social robots [2] are autonomous robots that are currently being developed on a large scale for safe and secure robot interactions in the human-centric environment [3]. The appearance and applications of these robotic systems vary; however, recognition in the context of object and place plays a central and vital role in these systems for semantic understanding of the environment. This article starts with the impact of social robots and lists the key features of some recently developed social robots that are tailored in public, domestic, hospital, and industrial use.

These robots are designed to interact and exhibit social behaviors with broad human-like capabilities, which integrate visual recognition, knowledge representation, task planning, localization, and navigation. Among all these, we focus on a systematic review of the approaches that address the most essential robotic capability, known as visual recognition. In this direction, we present data representation methods based on sensor modalities for 3D recognition using deep learning (DL) and examine the approaches for both 3D object recognition (3DOR) and 3D place recognition (3DPR).

Visual recognition is a vital component for robotic systems that operate in human environments. The methods to perform visual recognition tasks generally fall into two categories: either machine-learning-based approaches, which first require feature definition, i.e., using scale invariant feature transform [4], histogram of oriented gradients [5], and then classification techniques, such as support vector machine [6] or deep learning (DL)-based approaches that perform recognition task using convolutional neural networks (CNN) [7] without specifically defining the features.

Autonomous robotic systems deal with a large amount of real-world data. Therefore, the manually designed models of traditional machine learning algorithms are not feasible [8] for real-world robotics applications. On the other hand, the flexibility of DL-based models and their better performance as the scale of data increases make them well suited for use in robotics applications. Over the last few years, CNN-based DL models, starting in 2D space using two-stage [9,10] and one-stage object detectors [11,12,13,14,15,16], have achieved state-of-the-art object recognition results with the output of 2D bounding boxes (BBoxes).

Typically, two-stage detectors, such as R-CNN [17], Fast R-CNN [18], and Faster R-CNN [9], exploit region proposal networks in a first step to propose regions of interest (RoI). Afterward, they send region proposals to the network pipeline for object prediction by calculating features over RoI. As a trade-off for run time, one-stage detectors, such as YOLOv3 [15], YOLOv4 [19], Scaled-YOLOv4 [20], and single shot multibox detector [12] do not involve region proposal.

Researchers [12,15] have handled object detection as a regression problem and directly learned class probabilities to detect the object with bounding box coordinates. One-stage detectors are faster and capable of real-time performance; however, their accuracy rate is lower than two-stage detectors [21]. The task of place recognition is similar to object retrieval [22] and has been performed using dynamic object detection [23] or constructing object maps that contain object information in a place [24]. Although extensive research has been conducted on 2D recognition, it has potential limitations compared with 3D recognition.

With the recent monumental innovations in sensor technology, a wide variety of DL-based 3D object [25,26,27,28] and place recognition approaches [29,30,31] have been developed for different types of sensors. LiDAR and camera are two frequently used and increasingly popular sensors [32] that have been employed for object and place recognition in robotic systems. 3D object recognition predicts 3D information of objects, such as the pose, volume, and shape of the object with 3D BBoxes and class labels. It plays an important role in the intelligent perception of robotic systems.

In contrast to 2D object detection, it requires richer input data and efficient algorithms to estimate six degrees of freedom (DoF) poses [33] with high precision of oriented 3D BBox [34,35] dimensions for objects. 3D Place recognition involves distinguishing two identical places based on their sensor information [36]. Different approaches for place recognition are used, such as several feature maps that are correctly matched between images, learning representative features [37], and calculating the pixel-wise distance between camera images.

LiDAR-based methods for place recognition concentrate on developing local [38] and global [39] descriptors from structural information, segmenting [40] the point cloud (PC) data in 3D LiDAR point clouds and utilizing CNN techniques with 3D LiDAR PC by projecting range sensors on 2D images [41]. However, the synchronization of camera and LiDAR sensors [42] is essential for capturing detailed information of objects and large-scale place recognition.

### 1.1. Contributions

During the last decade, there has been rapid progress in the domain of social robots, including autonomous vehicles. Parts of this success rely on the implementation of both 3D object and place visual recognition tasks.

Previous reviews, shown in Table 1, concentrated only on 3D object recognition and did not address the 3D place recognition methods. In contrast to the previous studies, this article reviews and analyzes sensor-based data representation methods for both 3D object and place recognition (3DOPR) using state-of-the-art DL-based approaches. Moreover, we also discuss recently developed social robots.

This review is concentrated on 3D visual recognition approaches that have their applications in the domain of robotics, while approaches in the domain of smart environments are beyond the scope of the current survey. We aim at facilitating novice researchers and experts to overcome the challenging task of determining and utilizing the most suitable visual recognition approach for their intended robotic system, as one can quickly explore the recent research progress through this review.

Compared to the existing survey papers, shown in Table 1, the present review is different in the following terms, to the best of our knowledge:

- We discuss the latest representative social robots that have been developed recently (Section 2).
- The present study is the first article that comes up with a combined review of two robotic capabilities: 3D object recognition and 3D place recognition in a comprehensive assessment. It provides data representation modalities based on camera and LiDAR for both 3D recognition tasks using DL-based approaches (Section 3).
- It reviews 14 3D object detection datasets.
- The current survey presents a comparison of existing results to evaluate the performance on datasets.
- It yields an analysis of selected approaches from the domain of robotics, delineates the advantages, summarizes the current main research trends, discusses the limitations, and outlines the possible future directions.
- Compared to the earlier surveys, this study is more concerned with the most recent work. Therefore, it provides the reader an important opportunity to advance their understanding of state-of-the-art robotic 3D recognition methods.

### 1.2. Survey Structure

**Figure 1:**
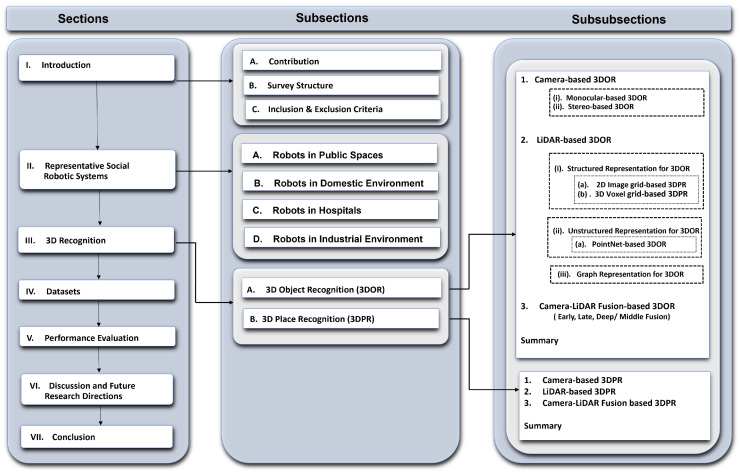
The overall structure of the survey that shows all the topics discussed in each section.

The survey has been organized in a top-down manner. The overall structure of the survey with corresponding topics and subsections is diagrammatically illustrated in Figure 1. In Section 2, the aim is to provide fresh insight to the readers into recently developed social robots with their impact on society, use cases, sensors, tasks (i.e., recognition), and semantic functions (i.e., assisting) in public places (Section 2.1), domestic (Section 2.2), hospitals (Section 2.3), and industrial environments (Section 2.4).

In Section 3, inspired by the recognition capabilities of social robots, as described in Section 2, the article examines the sensor (camera and LiDAR) based data representation approaches used for the 3D object (Section 3.1) and place (Section 3.2) recognition applying DL-based models. In addition, it gives a brief overview of datasets (Section 4) that have been used for the evaluation of 3D recognition methods. Consequently, in Section 6, the article discusses current research challenges and future research directions, and finally we conclude the survey with a summary in Section 7.

### 1.3. Inclusion and Exclusion Criteria

The inclusion and exclusion criteria are mainly focused on Section 3 for 3DOR and 3DPR methods. Section 2 does not involve comparison (instead it highlights the importance of visual recognition capability by giving the examples of recently developed robots from different sectors); therefore, it is not restricted to follow the same time span as Section 3. However, Section 3 performs the literature analysis for 3DOR and 3DPR methods; therefore, all studies in Section 3 are restricted to follow a specific time span based on inclusion and exclusion criteria. For 3DOR (Section 3.1) and 3DPR (Section 3.2), the inclusion criteria are as follows:

- The research publications must be from 2014 to 2021.
- Their domain must be a robotic system.
- They must be either journal or conference publications.
- They must address 3DOR or 3DPR methods using deep-learning approaches based on Camera and LiDAR sensor modalities.

Table 2 represents both inclusion and exclusion criteria that were applied to perform the paper selection, and the results of the systematic approach for paper filtering process are described below.

#### Results of the Paper Selection Process

**Figure 2:**
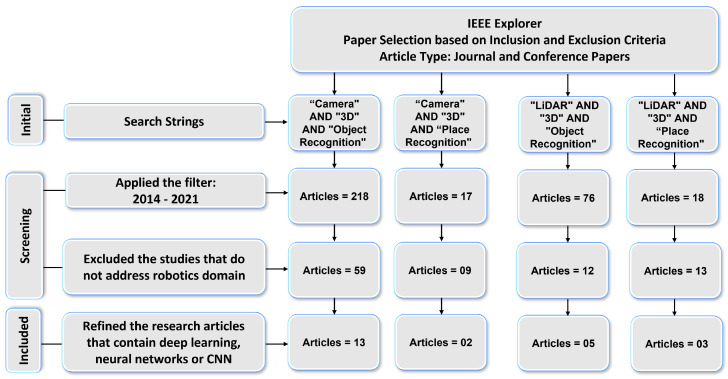
Results: IEEE Explorer paper selection based on the inclusion and exclusion criteria.

**Figure 3:**
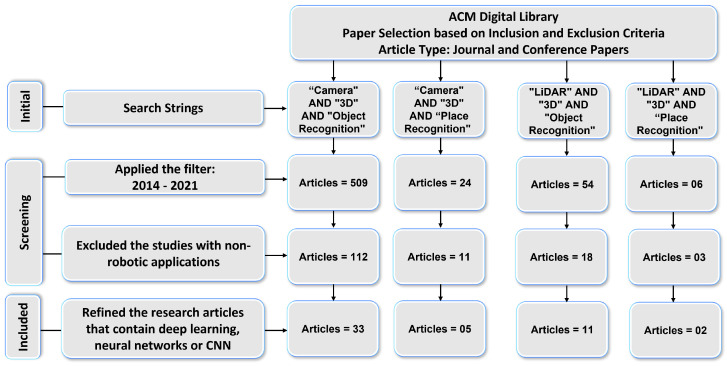
Results: ACM Digital Library paper selection based on the inclusion and exclusion criteria.

We conducted a systematic literature review for Section 3 to determine which DL-based models are being used for 3D object and place recognition based on sensor modalities. We used four search strings (“Camera” AND “3D” AND “Object Recognition”, “LiDAR” AND “3D” AND “Object Recognition”, “Camera” AND “3D” AND “Place Recognition”, and “LiDAR” AND “3D” AND “Place Recognition”) to extract the research articles from two key digital databases of academic journal articles that were IEEE Explorer and the ACM Digital Library. The paper selection process of this article consists of four steps as shown in Figure 2 and Figure 3.

First, the relevant articles for the survey from digital libraries using search strings were collected that correspond to the type of sensor (camera and LiDAR) and category of 3D recognition (object and place). In the second step, 329 articles in IEEE explores library and 593 articles in ACM digital library were extracted by applying the time period filter. The third step refined the 93 articles from IEEE Explorer and 144 articles from ACM Digital Library that belonged to the robotics category. We used MS Access database management software to find duplicates among these articles. For this, we ran SQL query on the database table and found that 35 articles in ACM and 21 articles In IEEE Explorer were duplicates.

After removing the duplicate articles, the fourth step involved splitting the articles that used deep-learning-based approaches and resulted in 23 articles from IEEE explorer and 51 articles from the ACM Digital Library that met the inclusion and exclusion criteria. Lastly, the selected articles based on their sensor data representation methods were arranged into 3DOR and 3DPR categories in which 17 articles from IEEE Explorer and 44 articles from ACM Digital library are related to the 3DOR task and five articles from IEEE Explorer and seven articles from ACM Digital library are related to the 3DPR task.

## 2. Representative Social Robotic Systems

This section presents recently developed social robotic systems that demonstrate recognition tasks and semantic understanding to perform a function in public (Section 2.1), domestic (Section 2.2), medical (Section 2.3), and industrial (Section 2.4) environments.

### 2.1. Robots in Public Spaces

Robots in public spaces indicates social robots used in places that are generally accessible for everyone, such as airports, supermarkets, libraries, and museums. Amazon launched a six-wheeled autonomous Scout delivery robot [45] in its Seattle-based research and development lab. It is commercially available in a few places in the USA, which are Atlanta, Georgia and Franklin, Tennessee after a long test run [90]. It uses an array of cameras and ultrasonic sensors for route planning and navigation on sidewalks at a walking pace and climbing up the front porch for package delivery. It has the ability of semantic task understanding, such as recognizing people and pets, detecting, and avoiding obstacles using machine learning algorithms.

AIMBOT [46] is an anti-epidemic autonomous driving robot that is designed for indoor crowded public environments, including schools, hospitals, and office buildings to provide safe and efficient Covid-19 protection. It is available for commercial use. It recognizes 200 people per minute, uses infrared thermal imaging camera to measures their body temperature, detects whether individuals are wearing masks, and sends a voice reminder to the people without a mask. Table 3 lists the sensors, purpose, and tasks as well as their algorithm, appearances, semantic functions, and development status.

### 2.2. Robots in Domestic Environment

Robots in the domestic environment refer to the robots that are used at homes for household chores, entertainment, or personal assistance. At the consumer electronics show 2020, Samsung showcased a robotic chef’s assistant [47], which consists of a pair of arms that mimic human gestures to cook the meal and performs the task on voice commands. It downloads the appropriate skills and performs the tasks, such as slicing by picking up the knife, pouring the ingredients, and mixing them. It is equipped with sensors and cameras and relies on AI and computer vision algorithms for the recognition task. The prototype of the Samsung chef robot was first unveiled at KBIS 2019 [91]. It is not available commercially.

Amazon’s Astro [48] is an Alexa-based home assistant robot that combines Alexa, computer vision, and AI software. It is a commercially available robot for home security, including a six-month free trial of Ring Protect Pro that allows saving videos in Ring’s cloud storage [92]. It obeys voice commands, such as follow me or go to a specific room. It performs face recognition to deliver items to a specific person. It acts as a family companion and entertains children by playing music. It cares for elderly people by reminding them to take medicine and record their blood pressure. It also assists to take voice or video calls. It uses SLAM for mapping the environment and roaming around the house. It automatically attaches itself to the charging dock. House members can use its mobile application for remote monitoring if they are outside.

Table 4 presents the sensors, usability, and tasks of domestic robots along with their algorithm, appearances, semantic functions, and development status.

### 2.3. Robots in Hospitals

Robots in hospitals are used in healthcare and treatment centers for relieving medical personnel either by aiding in surgery or caring for the patients. Moxi is a robotic assistant [49] in semi-structured hospital environments that is commercially available. The Medical City Dallas Heart and Spine Hospital is the first North Texas health care provider using the Moxi robot to combat a lack of nursing personnel in hospital systems [93]. It uses AI and machine learning algorithms to reduce the cognitive workload of nurses by performing tasks that do not require interaction with patients, such as delivering supplies to patient rooms, fetching items, and removing linen bags. Table 5 illustrates its characteristics, which include the robot’s sensors, purpose, and tasks, algorithm, appearances, semantic functions, and development status.

Ahn et al. [50], developed a multi-robot system consisting of ReceptionistBot and CareBot for the hospital environment that performs the tasks of receptionist, nurse assistant, and medical server. Both ReceptionistBot and CareBot are in the prototype stage and are not available commercially. ReceptionistBot communicate with patients and obtains their personal information. If visitors want to meet the medical staff, it guides them to meet CareBot for treatment. Carebot collects data about the patient’s health condition by asking questions. It assists the nurse using different healthcare devices to measure the blood pressure, pulse rate, and oxygen level of the patients. It also communicates with RoboGen, which is a secure server for managing patient information. MAiRA [51] is a multi-sensing intelligent robot that assists in complex medical procedures. This intelligent assistant is a commercially available cognitive robot [94]. It has voice recognition capability.

It performs human–robot interaction in a collaborative industrial environment. It can learn from instructions given through voice commands or gestures. It can perform object detection, pose estimation, and object grasping tasks either with professionals or wholly autonomously.

### 2.4. Robots in Industrial Environment

Robots in industry are used to assist in manufacturing by automating repetitive tasks, such as welding, assembly, and shipping. Handle is an autonomous mobile manipulation robot [52] developed by Boston Dynamics for moving boxes in a warehouse and unloading them from shipping containers. It relies on a 2D and 3D perception learning-based vision system to detect boxes. Table 6 enumerates the sensors, usability, tasks, algorithm, appearances, semantic functions, and development status. Handle will be available for sale in two years according to Playter the Chief Executive Officer at Boston Dynamics [96]. LARA [53] is a collaborative industrial robotic arm, developed recently. Its prototype is complete. However, it is expected to be realized soon for commercial use [97]. It is available in two sizes with 5 and 10 kg payload capacities. Its 3D vision allows detection and recognition of an object for a manipulation task.

Stretch [54] is a recently designed robot for autonomously moving boxes around the warehouses. Boston Dynamics expects that the robot will be commercially available from 2022 [98]. The strength of its arm makes it unique for potential entry into robotic warehouses. It is flexible and can do different tasks, such as loading, unloading boxes, and building up pallets.

## 3. 3D Recognition

With the recent breakthroughs in deep learning (DL) and significant improvements in sensor technologies, 3D recognition has made great progress, which leads toward rapid development in autonomous robotic systems, including autonomous driving. In this section, we concentrate on camera and LiDAR-based data representation methods employed for both 3D object recognition (3DOR) (Section 3.1) and 3D place recognition (3DPR) (Section 3.2) using DL models. Recently developed autonomous robotic systems (as described in Section 2) are mostly equipped with both cameras and LiDAR for visual perception tasks.

LiDAR is suitable to work with real-time autonomous systems in both indoor and outdoor environments, although most of the perception approaches focus on the use of LiDAR in autonomous vehicles. However, recent trends in deep-learning-based end-to-end approaches have also led researchers’ interest in the innovative use of LiDAR in autonomous robots for recognition tasks that benefit from the detailed 3D PC data to detect objects accurately. The PC data provided by the LiDAR sensor retains information related to the object’s position and reflection intensity as well as shape representation of different objects in complex scenes.

Hence, integrating this 3D PC information with DL-based recognition models is indispensable to perform precise 3D recognition. On the other hand, monocular and stereo cameras are less expensive sensors than LiDAR for 3D object detection but require post-processing techniques to determine the size and relative distance. The detection capability and reliability of the camera and LiDAR are limited in different environments. Table 7 summarizes the advantages and limitations of both sensors. Camera-LiDAR fusion is used to overcome these issues.

### 3.1. 3D Object Recognition (3DOR)

**Figure 4:**
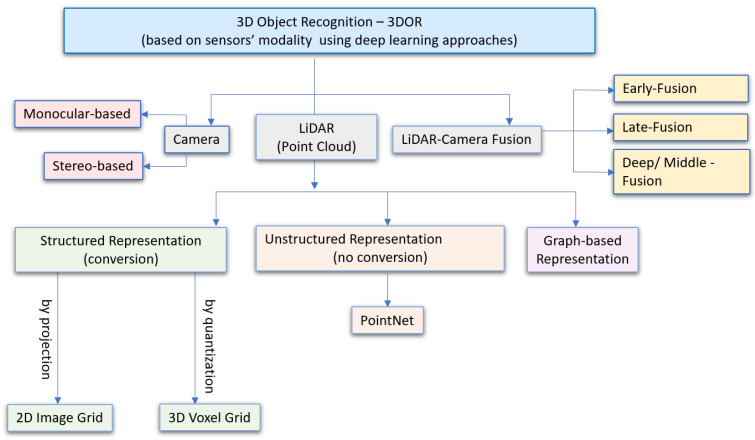
Camera and LiDAR-based Data Representation Modalities for 3D Object Recognition (3DOR).

This section categorizes data representation methods based on sensors’ modalities for 3D object recognition using deep learning in autonomous robotic systems. Compared with traditional recognition methods, the success of DL in the past ten years for robust and accurate object detection has made deep CNN the most promising method to perform 3D vision recognition tasks for robotic systems. The overall taxonomy is shown in Figure 4, which illustrates data representation in visual sensors that include a camera (Section 3.1.1), LiDAR (Section 3.1.2), and camera-LiDAR fusion (Section 3.1.3).

#### 3.1.1. Camera-Based 3DOR

This section explores the methods that perform 3DOR by estimating 3D bounding boxes (BBoxes) based on either monocular or stereo camera images as discussed in Table 8 with limitations and research gap. We first give an overview of camera-based methods and then describe their advantages and limitations in Table 9.

(i) **Monocular-Based 3DOR**

A monocular camera is essential for the deployment of low power and low-cost systems in the real-world application of robotics or autonomous driving [99]. Therefore, researchers have shown increasing interest in monocular 3D object detection in recent years [34,100,101,102,103,104]. Even though existing 3D detectors have achieved good accuracy, most of them do not consider the information related to occluded objects, which are partially visible. To this end, Chen et al. [55] improved 3D object detection by establishing a relationship of paired samples, which allows modeling spatial constraints for occluded objects. Its 3D detector introduced an uncertainty-aware prediction module for computing object location and object-to-object distances.

**Figure 5:**
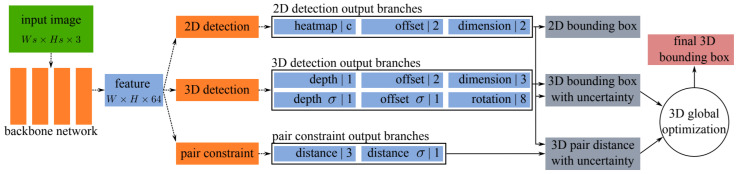
The architecture [55] overview with eleven prediction branches divided into 2DOR, 3DOR, and pair constraints.

This method adopted a one-stage architecture by sharing the anchor-free 2D object detection approaches, consisting of one backbone and several task specific dense prediction network branches. The backbone accepted one monocular image as input while (WxHx64) size as output feature map. It had eleven output branches as shown in Figure 5, which were divided into three parts: three for 2DOR, six for 3DOR, and two for the prediction of pairwise geometric constraints, which were estimated among adjacent objects using key points on the feature map.

**Figure 6:**
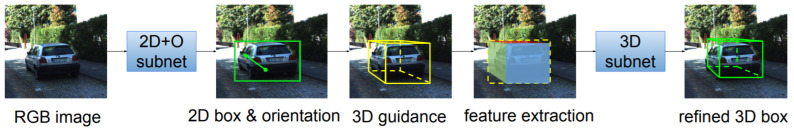
The proposed 3D object detection paradigm [56] consisting of a CNN based model (2D+O subnet), 3D guidance generated using the obtained output of 2D+O subnet, and extracted features utilized by the refinement model (3D subnet).

Li et al. [56] presented a 3D object detection method by extracting 3D information from a 2D image and generated accurate 3D BBoxes by obtaining coarse cuboids of predicted 2D boxes. In contrast to typical methods that rely on feature extraction from 2D BBoxes, it exploited 3D structural information by employing visual features and used the extracted features from surfaces to eliminate the feature ambiguity problem of 2D bounding boxes. It modified faster R-CNN for orientation prediction by including a new branch. Figure 6 shows an overview of its proposed framework in which single RGB image was passed as input, and it was processed in four steps. First, a CNN-based detector, known as 2D+O subnet, was used for extracting 2D BBoxes and orientations of the objects.

In the second step, these were utilized with the prior knowledge for driving scenario and basic cuboid were generated, which were called guidance. In the third step, this guidance was projected on the image plane and features were fused as distinguishable structural information to eliminate the ambiguity. In the fourth step, another CNN called 3D subnet was used fused features as the network input to improve the guidance.

**Figure 7:**
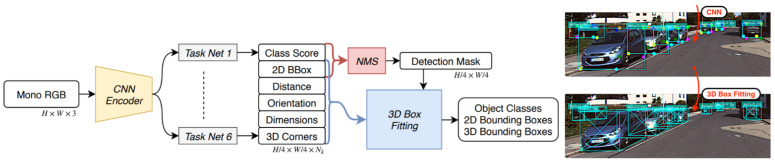
The pipeline of SS3D [57] for 3DOR from a single view.

Jörgensen et al. [57] proposed single-stage monocular 3D (SS3D) architecture. It contained two main parts: a CNN that was used for detecting the objects by regressing a surrogate 3D representation and a 3D BBox optimizer for fitting respective 3D BBoxes. SS3D regressed 2D and 3D BBoxes simultaneously after specifying the object’s center and its 2D and 3D BBox tuple contained 26 surrogate elements. Its proposed pipeline is illustrated in Figure 7 and consists of three steps. The first step is object detection with class scores and regression for 3D BBoxes’ fitting, while the second step involves non-maximum suppression for the elimination of redundant detections. Finally, 3D BBoxes were yielded through an optimizer using learning weights, and these 3D BBoxes were fitted independently and in parallel using the non-linear least squares method.

**Figure 8:**
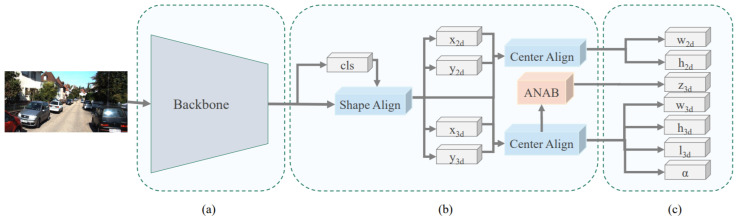
The architecture of M3DSSD [58] for monocular 3D object detection (**a**) Framework backbone. (**b**) The two-step feature alignment, classification and regression heads with ANAB for depth prediction. (**c**) Other regression heads.

Luo et al. [58] introduced a monocular 3D single stage object detector (M3DSSD) to overcome the feature mismatching issue of anchor-based monocular 3DOR methods by proposing a two-step feature alignment approach. The major components of its architecture shown in Figure 8 are a backbone network that is modified version of [105], feature alignment, attention block, and prediction head. Its asymmetric non-local attention block (ANAB) extracts depth-wise features for representing the global information. Its feature alignment consisted of two steps to handle the misalignment of 2D and 3D BBoxes. The first step obtained the target region based on the classification confidence and allowed the respective filed of the feature map to concentrate on the anchor regions. The second step used the 2D/3D center prediction for feature offset estimation to reduce the gap between predictions and feature maps.

(ii) **Stereo-Based 3DOR**

**Figure 9:**
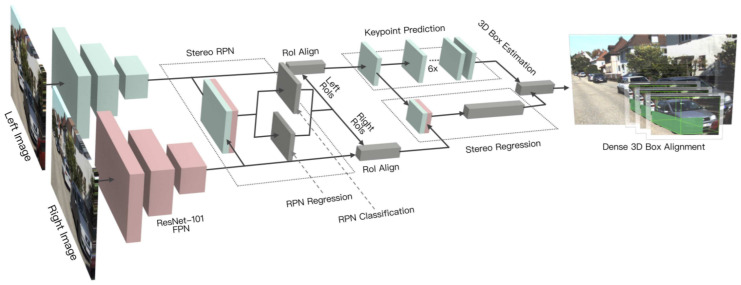
The architecture of Stereo R-CNN [59], which outputs key points, stereo boxes, along with the viewpoint angle and dimensions, followed by 3D BBox estimation.

Compared to the monocular camera, there are relatively fewer studies that utilize stereo vision for 3D object detection. Li et al. [59] exploited semantic and geometric information in the stereo image by proposing a stereo R-CNN based 3D object detector, which was an extension of Faster R-CNN. The stereo region proposal network, stereo R-CNN, and key points branch were three major components of its architecture as shown in Figure 9.

The stereo region proposal network module generated right and left RoI proposals. The stereo R-CNN module applied RoI-Align [10] on feature maps and concatenated them for object classification. It adds a stereo regression branch for accurate regression of 2D stereo boxes. The key point branch took left RoI features for detecting object key points. It performed 3D box estimation by projecting the relations between 2D right-left boxes with 3D box corners and key points. It specified accurate 3D bounding boxes and object localization by employing a dense region-based photometric alignment method.

**Figure 10:**
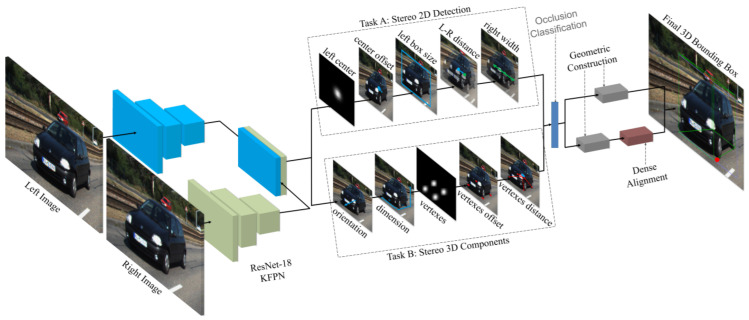
Network architecture of Stereo CenterNet [60] with 10 outputs and sub-branches for two tasks and the estimated 3D BBoxes.

Inspired by CenterNet [106] and Stereo R-CNN [59], Shi et al. [60] proposed a 3D object detection method to recognize the target by extracting semantic and geometric features in stereo RGB images without relying on depth information. It used 2D left-right boxes and predicted four semantic key points of the object’s 3D BBoxes while optimizing the position of 3D BBoxes using a photometric alignment module. Its network was built on CenterNet, which extracted the features from left and right image architecture as shown in Figure 10 using a weight-share backbone, which outputs 10 sub-branches. It performed two tasks. The first task is related to stereo 2D detection in which five sub-branches estimate the center, offset, and BBox of the left object. The second task is the stereo 3D component in which five sub-branches were used to estimate the dimension, orientation, vertices, and center distance of 3D BBoxes for left objects.

#### 3.1.2. LiDAR-Based 3DOR

LiDAR gives accurate depth information of the environment for 3DOR by discretizing the whole 3D space [107]. The major challenges toward applying DL-based approaches for LiDAR-based 3D object recognition research are the unordered, irregular, discrete, and sparse data representation of PCs, which makes it difficult to process point clouds data directly with CNN-based models. This is due to CNN models rely on convolution operation, which takes ordered, regular, and structured data. More recently, literature has emerged with different methods to address PCs data processing challenges using CNN for 3D recognition. This section divides DL-based 3D recognition methods for LiDAR point clouds into three categories: structured (ordered), unstructured (un-ordered), and graph-based representation.

(i) **Structured Representation for 3DOR**

This section discusses 2D image grid and 3D voxel grid-based representation for LiDAR-based 3DOD via deep-learning approaches.

(a) **2D Image Grid-Based 3DOR**

Much of the current literature on 3DOR pays particular attention to project discrete 3D PC data into a 2D grid representation using DL-based models. Table 10 gives a brief overview of the 2D image grid-based 3DOR method with current restrictions and research gaps. Studies along with their advantages and limitations are discussed in Table 11.

**Figure 11:**
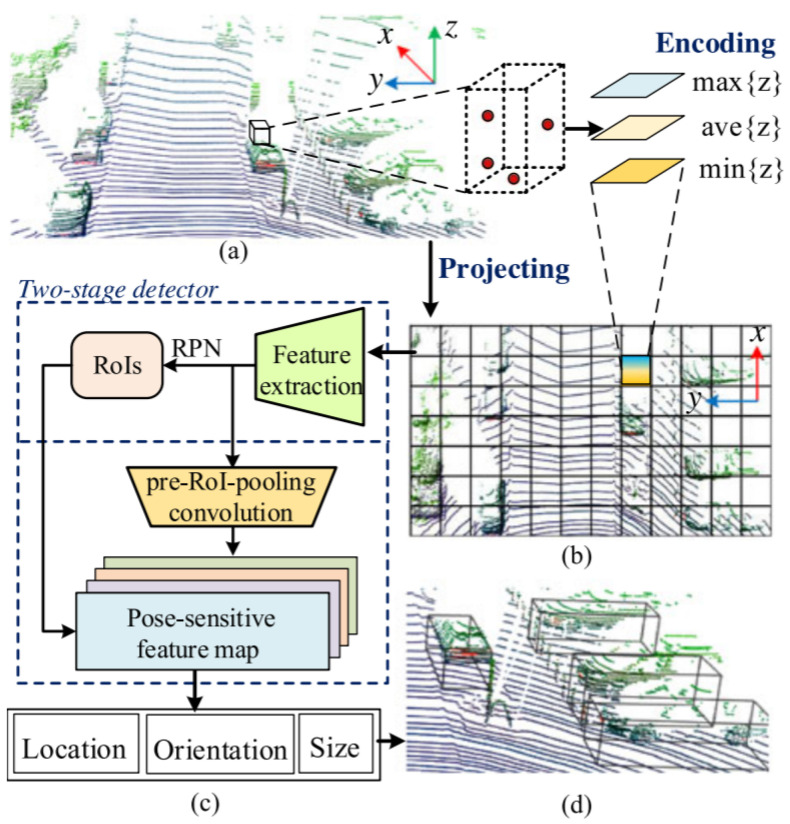
The pipeline of RT3D [61]: (**a**) LiDAR-based 3D point cloud on (**b**) a depth map encoded with height information of points; (**c**) a CNN-based two-stage detector is utilized for region proposals generation and their classification on pose-sensitive feature maps; (**d**) visualization of detected vehicles with orientated 3D BBoxes.

Zeng et al. [61] utilized pure LiDAR PC on a 2D grid and introduced a real-time 3D detection method RT3D illustrated in Figure 11 using two sub-networks: region proposal network and classification sub-network. Its pipeline contained three major steps. First, sparse 3D point clouds were projected on a 2D grid representation for converting them into the input format of CNN. After that, height information from point data was embedded in the 2D grid for 3D object detection. Thirdly, the 2D grid information was passed to a two-stage CNN detector, which generated region proposals.

This was initialized with pre-trained ResNet-50 model [108], while it adopted Faster-RCNN [109] techniques for the generation of region proposals on feature map and introduced pre-RoI pooling convolution techniques before RoI operations to improve the computation efficiency. Subsequently, classification and location regression for each RoI was performed to define the location, orientation, and size estimation with a pose-sensitive feature map. This addressed two problems related to the sparsity of PC: First, deleting empty anchors that contained no data on feature maps; Second: adopting automatic selection of hard examples using online hard example mining [110] to provide end-to-end efficient and effective network training.

Most PC-based 3D object detection methods use anchor-based detection methods, which have two major disadvantages. First, these methods require Non-Maximum Suppression (NMS) to filter redundant, overlapped, and imprecise bounding boxes (BBoxes), which causes non-trivial computational costs. Second, they require tricky anchor tuning, which is time-consuming.

**Figure 12:**
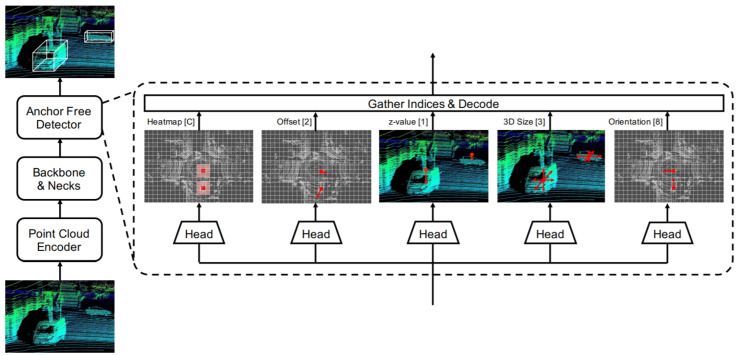
3D detection pipeline of AFDet [62]. The numbers in square brackets represent output channels of the last convolution layer, and C indicates the number of categories.

In this direction, Ge et al. [62] proposed AFDet, which is the first anchor and NMS-free PC 3D object one-stage detector with straightforward post-processing. Its 3DOR detection pipeline consisted of four major components, which were a point cloud encoder, the backbone, and necks, and it also included an anchor free detector as shown in Figure 12. It encoded PC to image-like feature maps in birds eye view (BEV) using [111]. Then, it used a CNN with up-sampling necks, which were connected to five different heads for the prediction of object centers in the BEV plane using key point heat map and regression of 3D BBoxes. It combined the head outputs to generate detection outcomes. Every heat peak was selected by a max pooling operation during the inference, which eliminated the need for NMS.

(b) **3D Voxel Grid-Based 3DOR**

Many LiDAR-based 3DOR techniques use a voxel grid representation [112]. Table 12 explains the brief methodology, limitations, and the research gap, and we summarize the reviewed models with advantages and limitations in Table 13.

**Figure 13:**
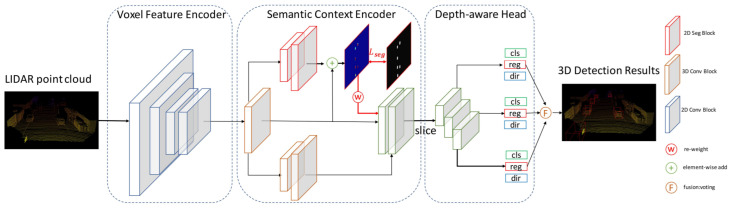
The SegVNet [63] with major components VFE, SCE, and depth aware head.

LiDAR PC-based 3D vehicle detection is important for obstacle avoidance in real-world robotics applications, such as autonomous driving. The semantic context information in LiDAR-based sensors is not deeply explored in the literature. Therefore, despite significant progress, vehicle ambiguity and the varying distribution of PC across different depths are two main problems. Yi et al. [63] addressed these issues by developing free-of-charge BEV semantic masks and a depth-aware learning head in the fully convolutional network. They proposed a one-stage detection framework, SegVNet, consisting of three major components: a voxel feature encoder (VFE), semantic context encoder (SCE), and depth-aware head as shown in Figure 13.

They introduced a VFE for voxelized feature representation of raw PC and developed a SCE for taking BEV feature maps from VFE as input and generated the semantic context encoded feature maps as output for 3D detection. SCE shared VFE feature maps with its two branches, in which, the first is adopted from [113], while the second learns BEV semantic masks predictions. Its depth-aware head consisting of convolution layers with different kernel sizes was designed for learning distinctive depth-aware features across different depths in autonomous driving scenarios.

Many recent PC-based 3D detectors are optimized for classes, such as cars, pedestrians, and cyclists with multiple models; therefore, it requires a large number of resources to run multiple models for obtaining the desired detection results, which are not desirable for autonomous driving vehicles that have limited resources. 

Muramatsu et al. [64] presented their solution by developing the SECOND-DX model to support multi-class LiDAR-based 3D object detection with only a single model in real-time. This extended the [113,114], and [111] models to provide support for three classes: cars, pedestrians, and cyclists. It divided the PC into a 3D spatial grid and extracted fine local features using a high-resolution voxel. It contained three sub-networks in which the first [111] was used to convert points to voxel-wise representations, the second sub-network improved the spatial feature map and encoded it to a 2D feature map, and class probabilities and direction classification were performed by the last region proposal network.

**Figure 14:**
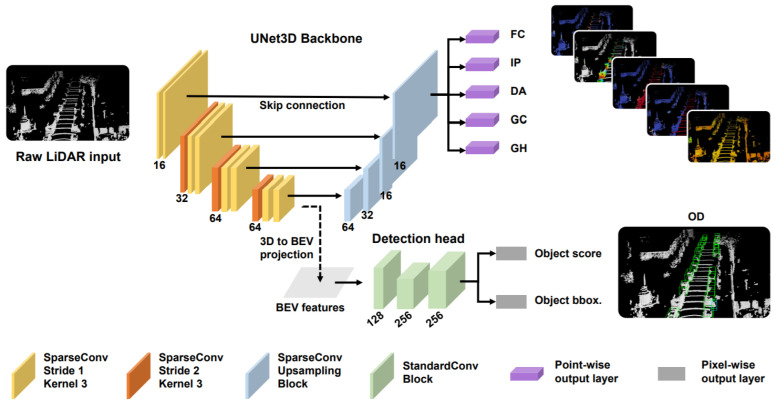
The network [65] is based on a UNet backbone with 3D sparse convolution and deconvolution to perform object detection on the Lidar BEV.

Feng et al. [65] proposed a LiDAR-based multi-task learning network (LidarMTL) to perform six perception tasks in a unified network for 3DOR. Its network architecture based on the voxelized Lidar point cloud is shown in Figure 14, which voxelized the 3D space into regular voxels. It well-preserved the geometric information by proper voxel size. It used UNet architecture to add task-specific heads and trained this entire network with multi-task loss. Following [115], they extended the encoder–decoder based [116] UNet architecture for efficient processing of 3D LiDAR points that were represented as voxels using 3D sparse convolution [113].

(ii) **Unstructured Representation for 3DOR**

This section focuses on Point-nets, and we analyze methods with their advantages and limitations in Table 14.

(a) **PointNet-Based 3DOR**

Point-nets directly handle the irregularities by taking raw LiDAR PC data as the input. This aims at reducing the information loss in 3D space caused by projection or quantization methods. Table 15 illustrates brief methodology, limitations, and the research gaps of pointNet-based 3DOR techniques, while Table 14 gives a literature analysis of the reviewed studies.

Most of the existing methods encode 3D PCs to 2D grid images by projection [73,117] or 3D voxel grid [114,118] and then apply CNN. However, the detection performance through these representations is not always optimal. Moreover, the limitation of these methods is their dependency on image detection results of 2D detectors, which do not give satisfactory performance in a large-cluttered environment. In a study, Yang et al. [66] addressed these issues by proposing an IPOD framework for 3D object detection on raw PC and provided a high recall rate. It seeded all points of cloud and object proposals without losing localization information.

**Figure 15:**
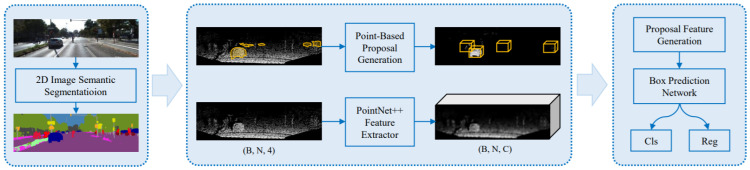
Illustration of IPOD [66] consisting of a sub-sampling network, point-based proposal generation, and the components of network architecture, which classifies and regresses the generated proposals.

It also extracted their local and context information, which was fed to PointNet for result generation through inference. It produced a 3D BBox from point-based object proposals and introduced the techniques for ambiguity reduction. Its network architecture shown in Figure 15 was consisted of a backbone network work based on PointNet++ [119], a proposal feature generation module with two parts for feature map extraction, and a BBox prediction network for the prediction of object’s size, shape, class, and orientation. It followed [114,120] to train one network for cars and the other for cyclists and pedestrians. 3D object detection from raw PC has been deeply investigated compared to other 3D detection methods.

**Figure 16:**
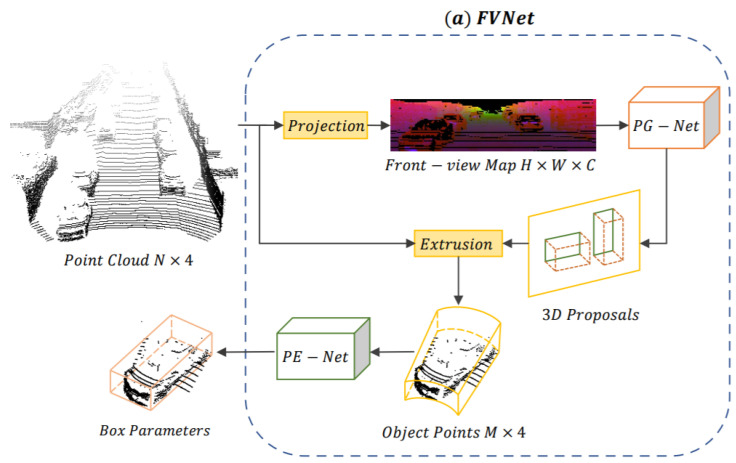
The network pipeline of FVNet [67] composed of PG-Net and PE-NET.

In a seminal study, Zhou et al. [67] presented an FVNet framework for raw PC-based 3D object detection and front-view proposals generation. Direct learning from PC is a challenging task due to its sparse and irregular points. The FVNet circumvented this issue by projecting raw PC on a cylindrical surface for front view feature map generation and took the advantage of both 2D image grid and 3D voxel grid while retained the rich information of 3D PC. The architecture of FVNet shown in Figure 16 was composed of two sub-networks. It used a proposal generation network (PG-Net) to predict the region proposals from the generated maps.

Then, these maps were used for the prediction of 3D region proposals. Finally, parameter estimation network (PE-Net), which extended the PointNet [121] structure, was used for the extraction of point-wise features and regression of 3D BBox parameters.

**Figure 17:**
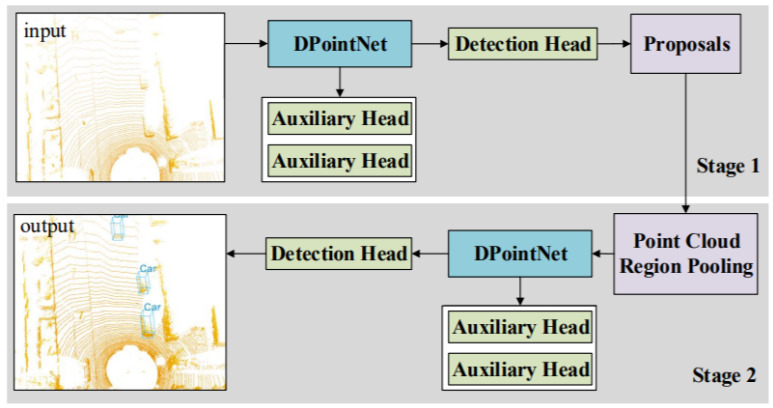
The architecture with DPointNet [68] detector consisting of two stages for 3D proposal generation and proposal refinement.

Li et al. [68] proposed density-oriented Point-Net (DPointNet) shown in Figure 17 to overcome the inhomogeneity of point clouds for 3DOR and verified its effectiveness on 3DOR by applying it to PointRCNN [122]. This network was proposed with two kinds of layers known as the SG (Sampling and Grouping) layer and several FA (Fusion and Abstraction) layers. It used the SG layer for sampling the seeds and their neighbors and several FA layers for fusion and abstraction of seeds features. The seeds from the input point cloud were sampled using farthest point sampling, and repeated random sampling was used if the neighbors were not sufficient.

The seed neighbors were divided into several groups according to the number of FA layers. Then, the next step was performed by FA layers, which used all neighbor information from SG layer. The FA layers were designed based on three schemes to fuse and abstract information for each seed. First, the feature appending scheme was used to transform the features of all groups in FA layer. Second, the coordinate concatenation scheme, was used to adopt the ‘concatenation’ mechanism for fusion using coordination information. Third, the feature concatenation scheme was used to combine first and second schemes by sufficient feature extraction and feature fusion. The auxiliary heads were applied to PointRCNN for training process.

(iii) **Graph Representation for 3DOR**

Graph-based representation preserves the irregularity of PC. However, only a few studies have investigated graph neural networks for 3D object detection in LiDAR PC. This section first discusses recent graph-based 3DOR methods and then analyzes them with their advantages and limitations as shown in Table 16.

Instead of converting PC data into grid or voxel representation, Shi et al. [69] proposed Point-GNN, a graph neural network for compact representation of PC in which neighbor-hood points were linked with the graph edges. It facilitated accurate detection of multiple objects on PC using 3D BBoxes in a single shot from LiDAR PC. The points were coordinated by the auto-registration method while detection results from different vertices and integrated by box merging and scoring operations.

Existing 3D object detectors individually recognize the objects without considering their relationship in learning and inference. The overall architecture contains three components. The first is graph construction in which a voxel down-sampled point cloud was used for reducing the density of a point cloud during graph construction. The second contained a GNN of T iterations in which a graph convolutional neural network was designed to refine the vertex’s state. The third was related to bounding box merging and scoring in which the merged boxes were calculated by considering the entire overlapped box cluster.

Feng et al. [70] presented a 3D relation graph network for building an object–object relation model by learning pseudo centers and direction vectors to improve the prediction accuracy. It was composed of two main parts in which 3D BBoxes were predicated through the proposal generation module, directly on the PC with PointNet++ [119] backbone. Its second part introduced the relation module for point attention pooling and exploit the object–object relationship.It also used point attention pooling for converting the point features into a uniform vector and performed relational reasoning using 3D object–object relation graph. It applied a 3D NMS post processing step for the extraction of high-quality 3D BBox candidates.

**Figure 18:**
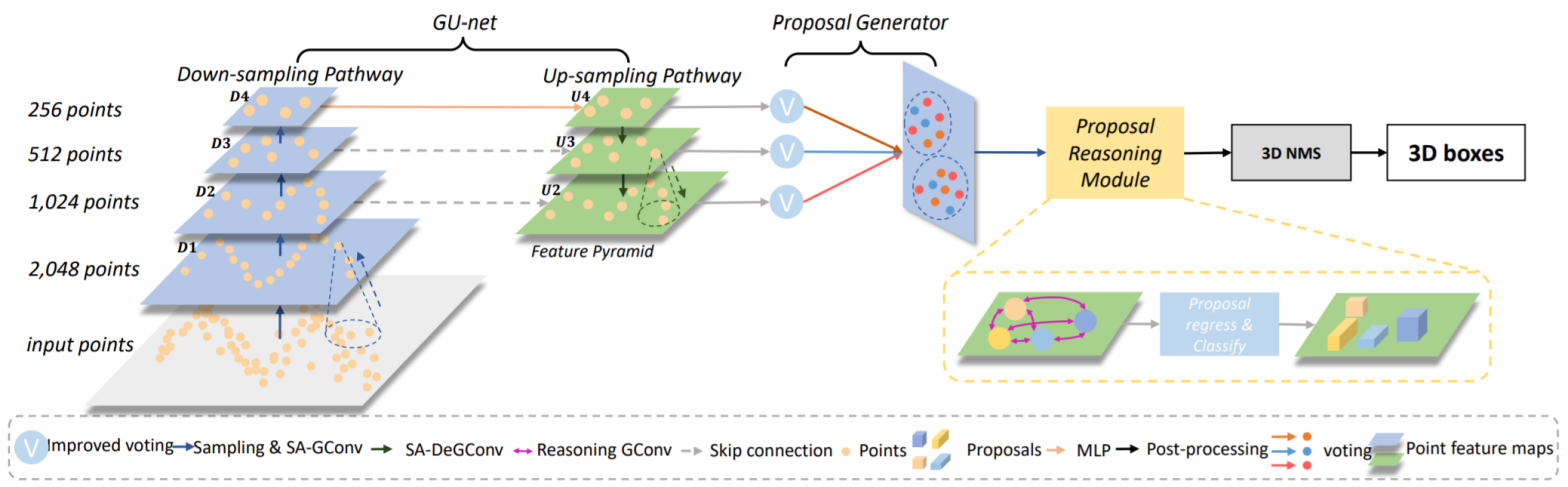
3D object detection pipeline of HGNet [71] framework with three main components: GU-net, Proposal Generator, and ProRe Module.

3D object recognition requires both geometric and semantic information (e.g., the object’s shape). However, many PC-based object detectors do not effectively capture the semantic characteristic of PCs. In this direction, Chen et al. [71] introduced the hierarchical graph network (HGNet) as shown in Figure 18 that processes raw PCs using multi-level semantics for 3D object detection. It contained three main parts, which are a graph convolution-based U-shape network called GUnet, proposal generator, and proposal reasoning module (referred to as ProRe Module).

It depicted the shape information of objects by extracting local features from geometric positions of the points. It employed a shape-attentive graph convolution, which is a U-shape network for mapping multi-level features through the voting module, and used ProRe Module to reason about proposals for BBox prediction by taking the advantage of global scene semantics. The proposal features were updated by GConv, combining the global scene semantics and including proposals’ relative positions as an attention map.

**Figure 19:**
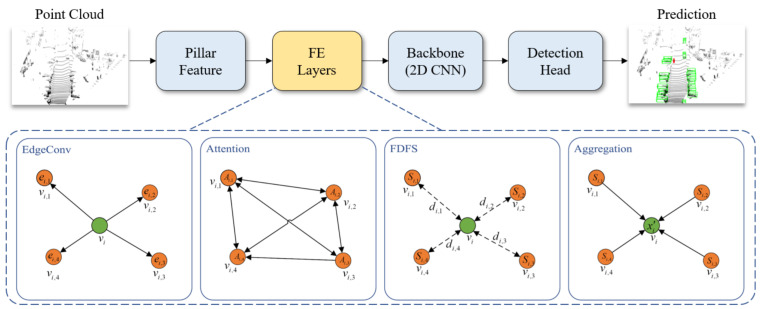
The pointPillars [72] with a feature enhancement layer.

Wang et al. [72] overcame the inherent drawbacks of partition-based methods that limit the 3DOR of small objects by proposing the spatial-attention graph convolution (S-AT GCN), which include EdgeConv, attention, far distance feature suppression, and aggregation steps as shown in Figure 19. For partition operation, single instance, e.g., a pedestrian was sliced, which is called the partition effect. The partition effect was used to influence the performance of 3DOR, particularly in the case of small object detection.

An extra layer called feature enhancement (FE) layer was included after partition operation. The S-AT GCN was cascaded to form FE layers, while the effectiveness of these layers was presented by adding [121]. They added the feature enhancement (FE) layer to the baseline model, point pillars [121] after partition operation and a spatial attention mechanism for GCN to extract geometric information. This enabled the network to extract more accurate foreground features.

#### 3.1.3. LiDAR-Camera Fusion-Based 3DOR

This section discusses 3D object detection based on camera-LiDAR fusion [123] using DL approaches to overcome the limitations and uncertainties of a single sensor. Camera-LiDAR fusion has become a practical approach for 3DOR [124]. The reliance on a single sensor can be risky for the accurate understanding of the surrounding environment, therefore, it is advantageous to equip robotic systems with a second sensor to achieve robust environment perception for the detection of 3D objects. To this end, sensor fusion, which leverages the data derived from multiple sensors and gives less uncertain information compared to the individual sensor, has become an emerging research area. Table 17 demonstrates the methodology and limitations along with the research gap of camera-LiDAR fusion-based 3DOR techniques.

The fusion approaches can be divided into three categories. Early fusion (EF), also called data-fusion, takes inputs from multiple sensors that are first combined in the beginning and makes a new representation that is used for transformations (e.g., convolutions). Late-fusion (LF), also known as decision fusion, first transforms the sensors’ inputs and then combines them. Deep-fusion (DF) or middle-fusion (MF) [125] is the combination of both EF and LF. We review some camera-LiDAR fusion methods and present their literature analysis in Table 18.

**Figure 20:**
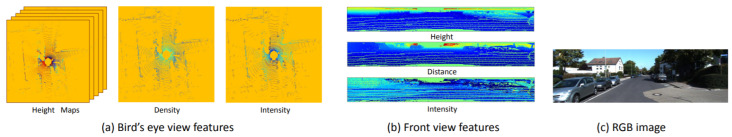
The input features of the MV3D [73] network.

Fusion approaches for 3D object detection are either very complicated or rely on late-fusion. Therefore, they do not provide multi-modalities interaction at the early stages. In this direction, Chen et al. [73] proposed multi-view representation of 3D (MV3D) point cloud, which included a bird’s eye view and front view of LiDAR and an image as input as shown in Figure 20. The representation of bird’s eye view was encoded by height, intensity, and density, while the complementary information was provided by the bird’s eye view representation. It was used for the fusion of both LiDAR PC and RGB camera images and the prediction of 3D BBoxes.

MV3D was composed of two sub-networks for the generation of 3D object proposals from BEV PC representation and fusion of multi-view features. It provided a deep fusion scheme after region proposal for combining region-wise features and enabled intermediate layer interaction. MV3D used 3D proposals to support different modalities and performed 3D box regression for accurate detection of objects, location, orientation, and size in 3D space.

**Figure 21:**
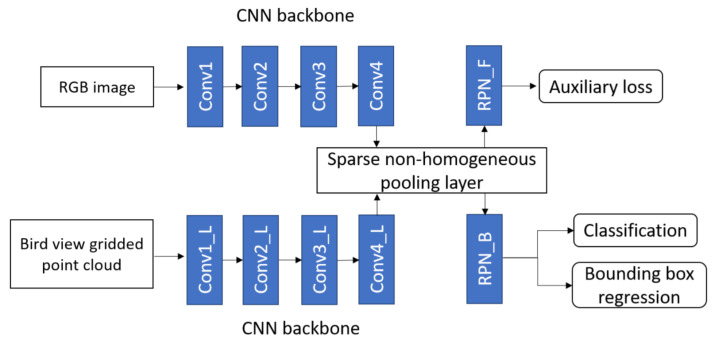
The fusion-based one-stage object detection [74] network.

Wang et al. [74] used deep CNN for camera-LiDAR fusion architecture to detect 3D objects in the autonomous driving scenario and efficiently transformed the features between BEV and front view by developing a sparse non-homogeneous pooling layer. The main idea to transform feature maps into different views by point cloud and matrix multiplication. A fusion-based network was built The network structure of one-stage fusion-based detection network was shown in the Figure 21, which contained two fully convolutional backbones for image and LiDAR units.

The PRN similar to many camera-based one stage detectors was used in image convolutional networks. However, region proposal was not used during the testing process. The auxiliary loss was applied to get supervision from the label and 3D proposal in the front view. It mapped two views by sparse PC and used a pooling layer to perform multi-view fusion before the proposal stage to transform the entire feature map. The architecture of its one-stage detector consists of two kinds of CNN backbone: VGG for camera-LiDAR with a feature map down-sampled four times for BEV and eight times in front view; MS-CNN [126] for camera-VoxelNet [114] with a feature map down-sampled two times for BEV and eight times in front view.

Roth et al. [75] performed deep end-to-end 3D person detection with a camera and LiDAR PC using deep CNN for estimating the 3D location and extent of people in the automotive scenes. Its architecture refined 2D anchor proposals by developing a region proposal network (RPN and subsequent detection network). It extracted high-level features from camera images using VGG-like CNN, obtained PC features through Voxel Feature Encoders [114], and performed end-to-end learning. The deep CNN learned low-level features from camera images and 3D LiDAR point clouds. It fused their high-level representations from both modalities and then passed them to the regression model as input for estimating the 3D person BBoxes.

**Figure 22:**
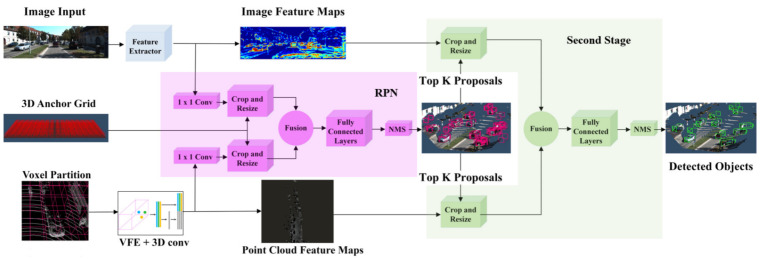
Aggregate view object detection [120] network pipeline for 3D object detection in the context of autonomous driving.

Figure 22 illustrated the network architecture, which was inspired by AVOD [120]. It adopted VGG16 network to extract the features of the image while features from the point cloud were extracted using voxel partitions. These partitions were applied by VFE layers and 3D convolutions. They size of the feature map was reduced by applying 1 × 1 convolution in RPN. The proposals were obtained by project 3D anchors on the feature map. The features from both modalities were fused after resizing and object’s location was estimated by applying fully CNN. In the second stage, the best proposal were cropped and fused from full feature maps. The fully connected layers for fused crops were used for the implementation of object detection layers. It allowed end-to-end network to the 3D locations of the persons from camera image and LiDAR point cloud data.

Sindagi et al. [76] extended VoxelNet [114] by introducing two fusion techniques: The point-fusion as an early-fusion scheme was employed to give a projection of PC to image feature space using a known calibration matrix, extract the features using a 2D detector, and perform point-level concatenation of image features. The voxel fusion as a late-fusion strategy was used to project non-empty 3D voxels generated by VoxelNet, extract features in 2D ROIs, and perform voxel-level concatenation of pooled features.

**Figure 23:**
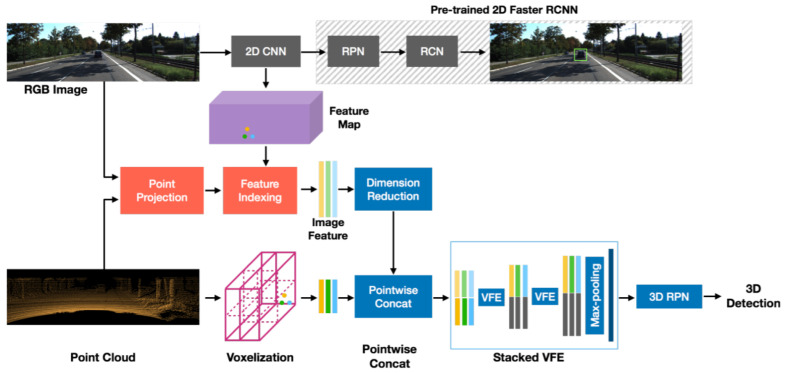
The overview of the MVX-Net [76] PointFusion method.

It was a later fusion technique to handle the empty voxels. The MVX-Net effectively fused multimodal information. Its PointFusion based method is illustrated in Figure 23 in which convolutional filters of faster RCNN were used to for extracting the image feature map. The 3D points on the image were projected by calibration information and related features were appended to the 3D points. The 3D RPN and voxel feature enhancement layers were used for the processing the aggregated data and 3D detections.

**Figure 24:**
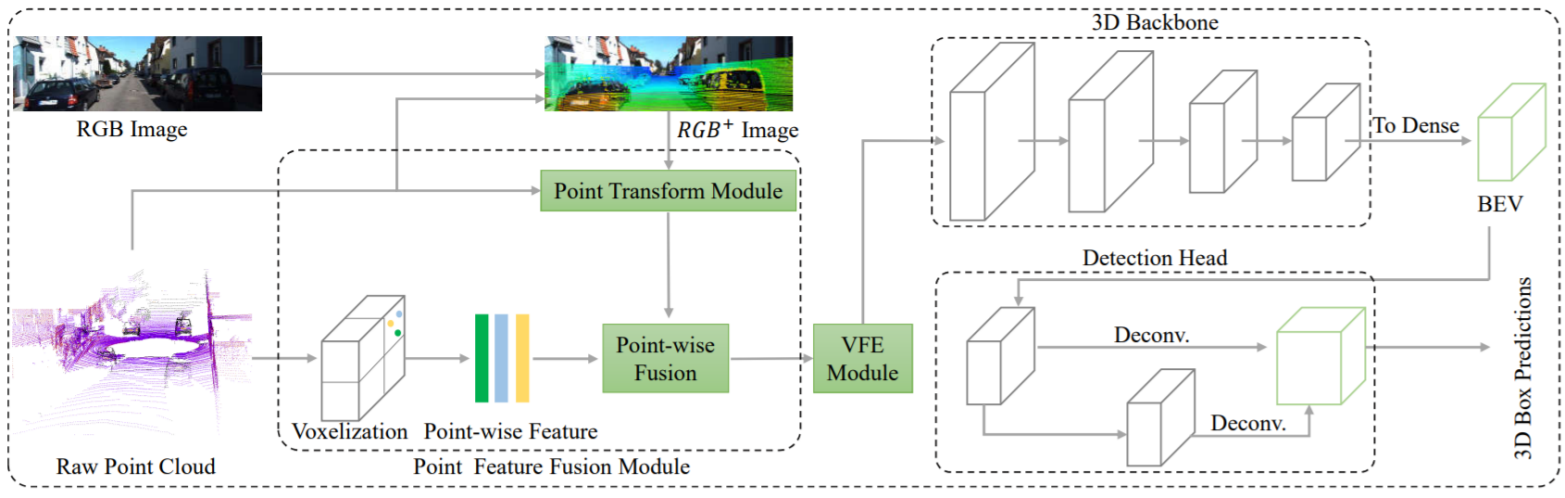
The pipeline of 3D object detection [77] network for the LiDAR and camera, including input, the point feature fusion module, the 3D backbone, and the detection head.

Wen et al. [77] proposed an early-fusion method to use both camera-LiDAR data for efficient 3DOR with single backbone network architecture. It extracted point-wise features from RGB images, which were fed into a 3D neural network. It used two strategies for reducing information loss during 3D voxel grid-based point-cloud representation. The first one was using small voxel size, while the second strategy was projecting point cloud features onto RGB images. A point feature fusion module, a voxel feature encoder module, a detection head, and a loss function were developed as the four main components of its one-stage 3D multi-class object detection model as shown in Figure 24.

The point clouds and RGB images were used as inputs and while the predictions of oriented 3D BBoxes for cars, pedestrians, and cyclists were the output. It used a point feature fusion module for the extraction of point features from the image and fused those features with the related point cloud features. High-level representation of fused point-wise features was performed by a voxel feature encoder module and 3D backbone and 3D BBoxes were classified and regressed by the detection head.

##### Summary

The summary of 3DOR according to the studies reviewed in Section 3.1 and listed in Table 9, Table 11, Table 13, Table 14, Table 16, and Table 18 is presented. Current applications of 3DOR are generally categorized into two environments: outdoor and indoor, with the first category being more frequently studied (19 vs. 4 studies). The article divides these 3DOR studies according to sensor modalities that include camera-based (monocular—five studies and stereo cameras—two studies), image grid-based (two studies), 3D voxel grid-based (three studies), pointNet-based (three studies), graph-based (four studies), and camera-LiDAR fusion-based (five studies). These 3DOR methods use state-of-the-art DL-based object recognition networks that follow either one-stage (nine studies) or two-stage (14 studies) object detection pipelines.

The advantages and limitations of 3DOR methods show that developing DL-based multi-model recognition systems is a particularly challenging task for ADV in outdoor environment because it requires a high level of accuracy and real-time performance while current models cannot generate prediction consistency over time. On the other hand, object recognition is a challenge in an indoor environment consisting of cluttered scene with many occluded objects. In addition, the fusion of multiple sensors and different feature representations as well as optimal fusion architecture for 3DOR are still open questions that require more focus on these research topics.

### 3.2. 3D Place Recognition (3DPR)

3D place recognition is a task of identifying the location in a view of a place by querying the similar images that belong to the same location in a large geo-tagged database [127]. It retrieves the database images according to the robot pose and current query image taken by the robot’s sensor (i.e., camera) to find the association between query images and database images of known places. Robots and automated vehicles on the road use the place recognition approaches for accurately recognizing the locations and efficiently identifying the revisited places.

Although, place recognition systems can also benefit from the existing research on object recognition by detecting the objects in the context of scene knowledge [128]. However, place recognition approaches are more concentrated on larger scale targets called the place landmarks [129]. Another major characteristic that distinguishes the place recognition from other visual recognition tasks is that it has to perform the condition-invariant recognition to a degree that many other recognition tasks do not have. Moreover, an architecture that is apt for 3DOR may not fit well into 3DPR tasks because their visual cues are different.

Place recognition is an active research area and a key capability of autonomous mobile robots. However, it is still a challenging task to achieve. The recent literature on place recognition concentrates on replacing traditional handcrafted feature extractors [4,130,131,132,133,134,135,136,137] with CNN for feature extraction [138,139,140,141], which aids in the direct learning of 3D structural descriptors. Camera and LiDAR are two main sensors to perform place recognition tasks.

Camera-based place recognition methods contain efficient descriptive information, but they struggle to cope with illumination and occlusion problems [142]. LiDAR-based place recognition approaches are invariant to appearance change [143], however, rich descriptive representation is still an open research question for LiDAR-based place recognition, and it suffers from limited ranging and motion distortion issues [114,144,145] Therefore, fusing information from both sensors provides better solutions.

This section reviews data representation methods for 3D place recognition based on Camera and LiDAR sensors using DL models. It is subdivided as Camera-based 3DPR (Section 3.2.1), LiDAR-based 3DPR (Section 3.2.2), and Camera-LiDAR Fusion-based 3DPR (Section 3.2.3).

#### 3.2.1. Camera-Based 3DPR

Visual place recognition (VPR) is the problem of recognizing a place from the robot’s current camera images based on the visual appearance [146,147]. It has been around for many years. However, research in this field is growing rapidly due to recent developments in camera technologies [148] with their compatibility for DL-based techniques. In this direction, 3D depth vision cameras and event-based cameras have drawn researchers’ attention. 3D depth cameras have made it possible to collect 3D data with ease. However, the limited range of depth, less accurate distance information, and training 3D data with DL-based models are the challenges still underdeveloped [149].

As DL-models rely on the networks trained only on RGB data, which lacks the depth features. In this direction, Song et al. [78] addressed these limitations using RGB-D videos for taking advantage of the richer depth and RGB information. It introduced a two-step training approach that involves weekly pre-training via patches to learn powerful depth-specific features. Its proposed CNN-RNN framework was used to model RGB-D scenes for recognition.

**Figure 25:**
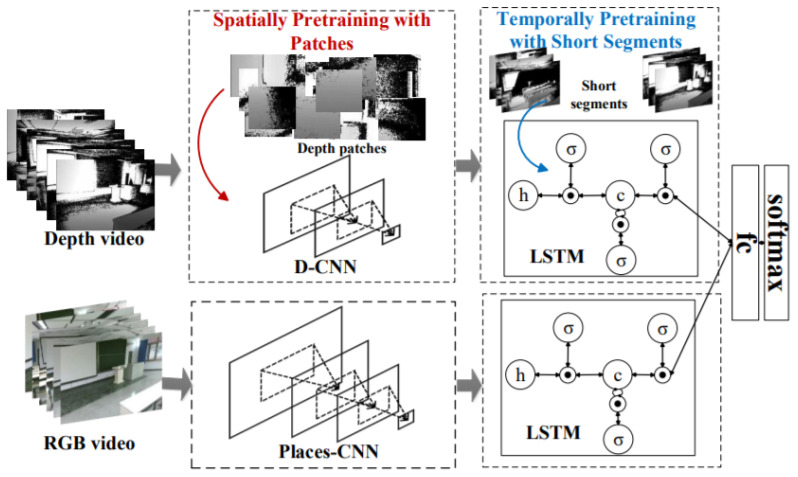
The CNN-RNN [78] architecture for video recognition.

Inspired by the two-step CNN techniques that were trained on still images, a three-step training strategy was introduced for CNN-RCNN architecture to obtain the significant gain through the integration of depth videos. It created a joint embedding by combining convolutional and recurrent neural networks for capturing spatial and temporal information as shown in Figure 25. LSTM blocks were used to implement the recurrent neural networks. It used independent branches for RGB and depth data. LSTMs based temporal embedding was modality specific and late fusion was performed using fully connected layer while combined architecture was trained jointly end-to-end.

**Figure 26:**
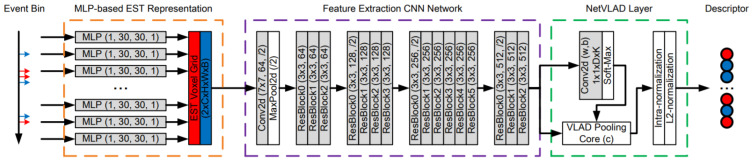
The pipeline of Event-VPR [79] for 3DPR.

To the best of our knowledge, there are very few studies that use an event-based camera for place recognition. Among them, Kong et al. [79] proposed Event-VPR, the first end-to-end VPR method using an event camera. These cameras work differently from the frame-based cameras because there are neuromorphic visual sensors that are inspired by the biological retina and have the advantage of low latency, low bandwidth and low power consumption [150]. The key idea of Event-VPR, as shown in Figure 26, was to apply NetVLAD to EST voxel grid, which was generated by event streams.

It selected the corresponding positive and negative of event bins and trained the network to learn the global descriptor vectors of the bins. First, it used event streams as input and divided the consecutive event stream into the bins. These bins were converted into EST voxel grid using MLP-based kernel. Then, the visual features of EST voxel grids were extracted using ResNet34 [108]. Then, feature descriptor aggregation was performed by a VLAD-based aggregated description layer, and finally the network was trained with weakly supervised training for 3DPR.

#### 3.2.2. LiDAR-Based 3DPR

Place recognition using LiDAR-based 3D PC is still an open issue and a harder task in large-scale dynamic environments due to the difficulty in feature extraction from raw 3D PC and global descriptor generation [151]. The article focuses on recent LiDAR point-cloud-based methods for 3D place recognition using DL-based techniques and provide their comparison in Table 19.

In contrast to image-based counterparts, most studies of 3D recognition have not dealt with LiDAR PC for place recognition due to the difficulty of its local descriptors’ extraction that can later be converted into global descriptors. A recent study by Angelina et al. [80] applied DL networks and introduced PointNetVLAD to provide the solution of PC-based place recognition using NetVLAD [152] and PointNet [121]. It extracted more general global features proposing lazy triplet and quadruplet loss function while mapped 3D PC to discriminative global descriptors by training PointNETVLAD using metric learning [153].

**Figure 27:**
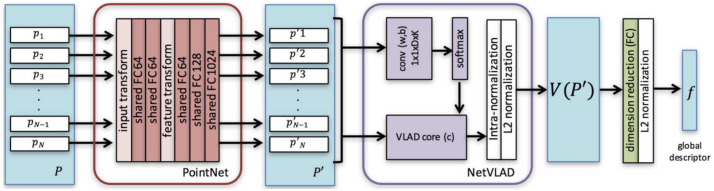
The architecture of our PointNetVLAD [80] network.

The PointNetVLAD was a combination of existing PointNet [121] and NetVLAD [152], shown in Figure 27 for global descriptor extraction from given 3D point clouds by end-to-end training and inference. Its included first block of PointNet that was cropped before maxpool aggregation layer. Its input was the same as PointNet consisting of a set of 3D points. The dimensional local feature descriptors were extracted from each input 3D point. These descriptors were fed to NetVLAD layer, which was designed to aggregate local image features from VGG/AlexNet into global descriptor vector. The VLAD descriptor [154] was the output of the NetVLAD layer.

Place recognition and scene understanding is also an important area of research in the indoor environment. However, in contrast to the outdoor environment, there are fewer studies of place recognition from 3D PC data for the indoor environment. An autonomous robot must be aware of different places, such as rooms, hallways, and kitchens in an indoor environment to perform its task. Huang et al. [81] performed 3D PC (voxel) based scene recognition in an indoor environment by combining semantic segmentation with the multi-task framework. It worked on scene recognition in indoor environment as supervised classification using neural network.

The network was composed of encoder to extract feature representation from input scene and a classification head to obtain class-conditional likelihood. It explored two different options for encoder: First was the working with subsampled version of original PC networks (Pointnet [121], Pointnet++ [119] DGCNN [155]) while second was sparse voxel grid networks (Resnet14 [108]). It demonstrated that multi-task learning with semantic segmentation improves the performance of scene recognition by sharing information among related tasks.

**Figure 28:**
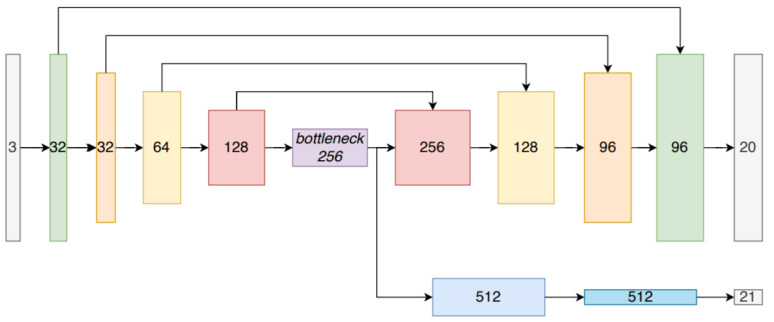
The multi-task network structure [81] for scene recognition in indoor environments.

The multi-task network was composed of an encoder for converting the scene into a feature representation, and two output heads, which were semantic segmentation head (top) and a classification head (bottom) for computing the class likelihood as shown in Figure 28. For semantic segmentation, sparse Resnet14 variant with U-net style decoder was extended that mirrored the encoder with skip connections. The network weights of encoder were froze and only scene classification head was trained. Finally the network was fine-tuned with small learning rate to yield better recognition.

**Figure 29:**
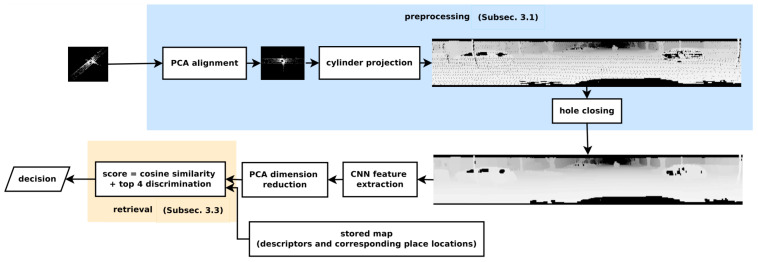
The system overview [82] for point-cloud-based place recognition using CNN feature extraction.

An efficient place recognition system is invariant to illumination variation and object motion in that place [156]. Sun et al. [82] presented PC-based place recognition using CNN that was pre-trained on color images and provided robust detection to moving objects, which were also rotation and illumination invariant. The 3D place recognition system in Figure 29 shows that it first aligned the PC with its principal directions then represented it onto the cylindrical image plan. It performed feature extraction using CNN followed by the principal component analysis dimension reduction and specified a threshold to determine the trade-off between recall and precision.

In its preprocessing step, it considered a 3D PC created by a Velodyne LiDAR to cover for full 360° environmental view. PCA was used to align the PC by finding the orthogonal directions and obtain more compact features. It generated the range image through the projection of PC on cylindrical plane while extracted the features by CNN using convolutional layers. It used fully connected layers to perform reshaping and pooling layer on the top of hidden layer for dimension reduction. Since one place contained one descriptor; therefore, the variance of dimension indicated its discrimination ability. For retrieval, the descriptor vector of each PC was normalized, and the cosine distance was used as similarity metric.

**Figure 30:**
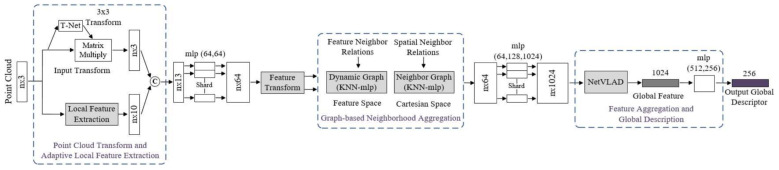
The LPD-Net [83] for large scale place recognition.

Liu et al. [83] proposed a large-scale place description network (LPD-Net) for extracting distinct and general global feature descriptors from 3D PC. It used local features rather than isolated point positions as the network input. The network architecture was composed of three major modules to handle large scale environment as shown in Figure 30. The adaptive local feature extraction module was used to obtain the PC distribution and the local features. The graph-based neighborhood aggregation module was used in feature and Cartesian space to learn structure information of PC. The resulting vectors were passed to NetVLAD [152] for the generation of a global descriptor.

The computational and storage complexity was reduced by extracting global descriptor to perform real-time place recognition tasks. Its feature network captured the local structure using features around each point in the local neighborhood. The raw PC data was passed as input to Transformation Net [121], which aimed at ensuring the rotational translation invariance and the adaptive local feature extractor, which considered the statistical local distribution.

The appropriate neighborhood size in different situations was selected using adaptive neighborhood structure, which were merged into feature vectors. The output of the feature network was passed to a graph network as input, and feature aggregation was performed using the kNNgraph network in the Cartesian space. It introduced the relational representation from the GNN to LPD-Net for representing the scene compositions as graph nodes, their intrinsic relationships and scene descriptors generated by GNN.

Most research on place recognition [80,157,158] has not fully addressed the problem of 3 DoF transformation. Schaupp et al. [84] dealt with the aforementioned issue by proposing an efficient data-driven framework for extracting compact descriptors from 3D LiDAR PC using CNN, which aimed at recognizing the place and regressing the orientation between point clouds. The network was trained by a triplet loss function and a hard-negative mining scheme was applied to improve the descriptor extractor. It developed a metric global localization in the map reference frame from single scan of 3D LiDAR PC.

**Figure 31:**
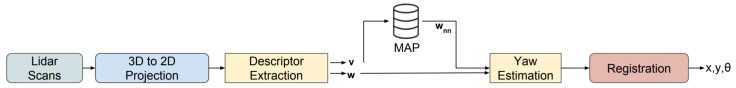
The methodology [84] of oriented recognition from 3D point clouds.

For this, it used four sequential components known as point cloud projection, descriptor extraction, yaw estimation, and local point cloud registration as shown in Figure 31. In the first step, PC projection used spherical model for PC representation and converted the LiDAR point cloud scan onto a 2D range image. In the second step, descriptor extraction was implemented for place representation and deriving orientation details using CNN.

For this, 2D range images were taken as input and two compact descriptor vectors were generated, which were used to represent rotation invariant and encode it for yaw angle discrepancy between the query PC and the PC of the nearest place in the map. Finally, local registration method was applied to obtain three DoF pose estimation using planar coordinates and orientation estimate. The deep CNN architecture based on [159,160] learned mapping from range image through encoding 3D PC onto feature vector representation to effectively perform oriented place recognition.

**Figure 32:**
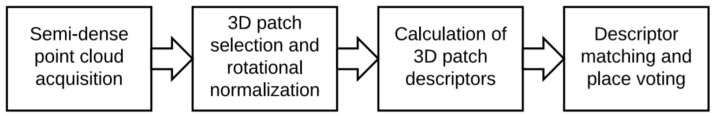
The pipeline of [85] place recognition pipeline in semi-dense maps.

Robust place recognition can be achieved using 3D scene structure. Ye et al. [85] represented structural information of the scene with semi-dense point clouds using DSO [132] and developed local descriptor matching to perform place recognition. It used 3D CNN like [118,161] and generated discriminative descriptors by learning features from a 3D-voxel grid. Its place recognition pipeline as shown in Figure 32 was composed of four main components. It used DSO [162] to acquire the information in semi-dense point cloud. It extracted the local patches from semi-dense point clouds and normalized them.

In the next step, keypoints were selected from random 5% resulting points and local cylindrical patches were extracted from them, which were chosen with the size to be as small as possible. These patches were represented using CNN-based descriptors, which contained two 3D convolutional layer, ReLU, a pooling and two fully connected layers for mapping from voxel grid to 512-dimensional descriptor. Finally, the resulting descriptors were matched to the descriptors that were stored in the database and their matches were aggregated to keyframe matches. It also used PCA to reduce the dimensionality, which resulted in efficient matching.

**Figure 33:**
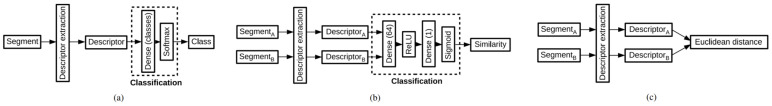
Three network structures [86] (**a**) group based CNN (**b**) Siamese CNN, and (**c**) descriptor extraction CNN trained using contrastive loss.

Cramariuc et al. [86] used segment extraction combined with a matching method to perform the place recognition task in LiDAR-based 3D point clouds. It used CNN to generate descriptors for 3D PC segments and introduced a segment recognition approach based on learned descriptors, which outperformed the SegMatch descriptors [163]. It extended the structures of [164,165] to the 3D domain for generating learning-based descriptors. It implemented place recognition task using three different CNNs as shown in Figure 33 for generating descriptors for 3D point cloud segments.

**Figure 34:**
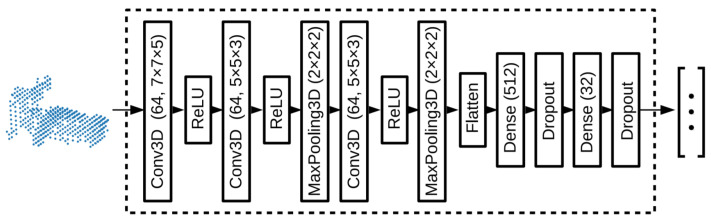
The descriptor extraction network [86] used in the three CNNs.

For preprocessing, the alignment method was chosen to increase the robustness and make the extraction process less sensitive. The augmentation techniques were used to make multiple copies of the segmented data by rotating each image at different angles. Then, the segments were scaled to fit and centered inside the voxel grid. A CNN was proposed for feature extraction. Figure 34 shows the structure of descriptor extraction CNN, which tested different depths and sizes for layers and filters to keep the network small enough it could be feasible to run on the mobile robot platform. The amount of dropout in the final layers was tuned separately to ensure a correct regularization.

The first approach was group-based classification. In this approach, training the CNN for segment classification was based on the groups that represent the classes. The layer before the classification was used as descriptor [166]. The closeness between the descriptors of segments of same group in the Euclidean space was loosely enforced by the classification layer. The probability of a segment belonging to a class was considered proportional to the dot product. The descriptors with small Euclidean distance were classified belonging to the same group. The candidate matches were generated by correlation between similarity and Euclidean distance between descriptors. The network was trained using SGD for minimizing the categorical cross-entropy.

The second approach was training a Siamese convolutional neural network [167] in which two inputs were passed to two distinct CNNs. These two CNNs were considered as two identical descriptor extraction networks. Then, the combination of output of two networks was given to third network, which generates the final output. The advantage of Siamese over two stage detectors was that it allowed training of feature extraction simultaneously. Feature extraction and classifier were used independently during the inference process to boot the performance. It also used GSD for training to reduce the binary cross entropy of the network.

The third approach was training the classifier with contrastive loss [165] for minimizing the Euclidean distance between the matching vectors while maximizing it for non-matching pairs. It recalculated the hard pairs (which had lowest Euclidean distance between their descriptors but the segments did not match and vice versa) at the end of each training epoch to increase the performance and avoid the local minima.

**Figure 35:**
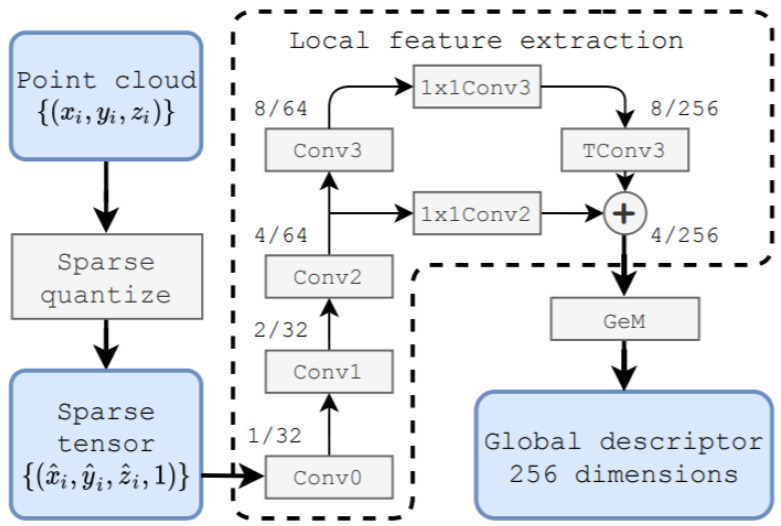
The network of MinkLoc3D [87] for point-cloud-based place recognition.

Komorowski et al. [87] used 3D FPN [168] and sparse voxelized point cloud representation inspired by MinkowskiNet [169] to propose discriminative 3D point cloud descriptor for place recognition. The local feature extraction network and generalized mean (GeM) pooling [170] layer were the two main parts of its network architecture as shown in Figure 35 for PC-based place recognition. A set of 3D point coordinates was passed as input and quantized into a sparse, single channel tensor. It used 3D Feature Pyramid Network [168] for local feature extraction. The GeM, which was the generalization of global max and average pooling, was used for the generation of global descriptor vector.

The network model was composed of four convolutional blocks that were used to generate sparse 3D feature maps and transposed convolution at its bottom-up and top-down parts, respectively. The top-down part was aimed at generating the upsampled feature map, which used lateral connection for concatenating with the features from the layers of bottom-up. It was intended to produce a feature map with a large respective field and high spatial resolution.

The bottom-up blocks from convolutional layer 1 to layer 3 were contained stride of two for decreasing the spatial resolution followed by residual block. batch normalization [171] layer and ReLU non-linearity were used for all layers in bottom-up blocks. Two 1x1 convolution blocks were aimed at unifying the feature maps channels of bottom-up blocks before they were concatenated in a top-down pass.

#### 3.2.3. LiDAR-Camera Fusion-Based 3DPR

This section reviews the methods that use fusion networks to generate global fusion descriptors based on camera-image and LiDAR PC for robust place recognition.

Xie et al. [88] presented the camera-LiDAR sensors fusion method, which robustly captures data from both sensors to solve the 3D place recognition problem. It introduced a trimmed clustering approach in 3D PC to reduce unrepresentative information for better recognition. They also built a compact neural network for robust representation of visual descriptor and 3D spatial global descriptor. It utilized deep neural network-based metric learning to minimize the distance of fused descriptors and to distinguish the similar and dissimilar places.

**Figure 36:**
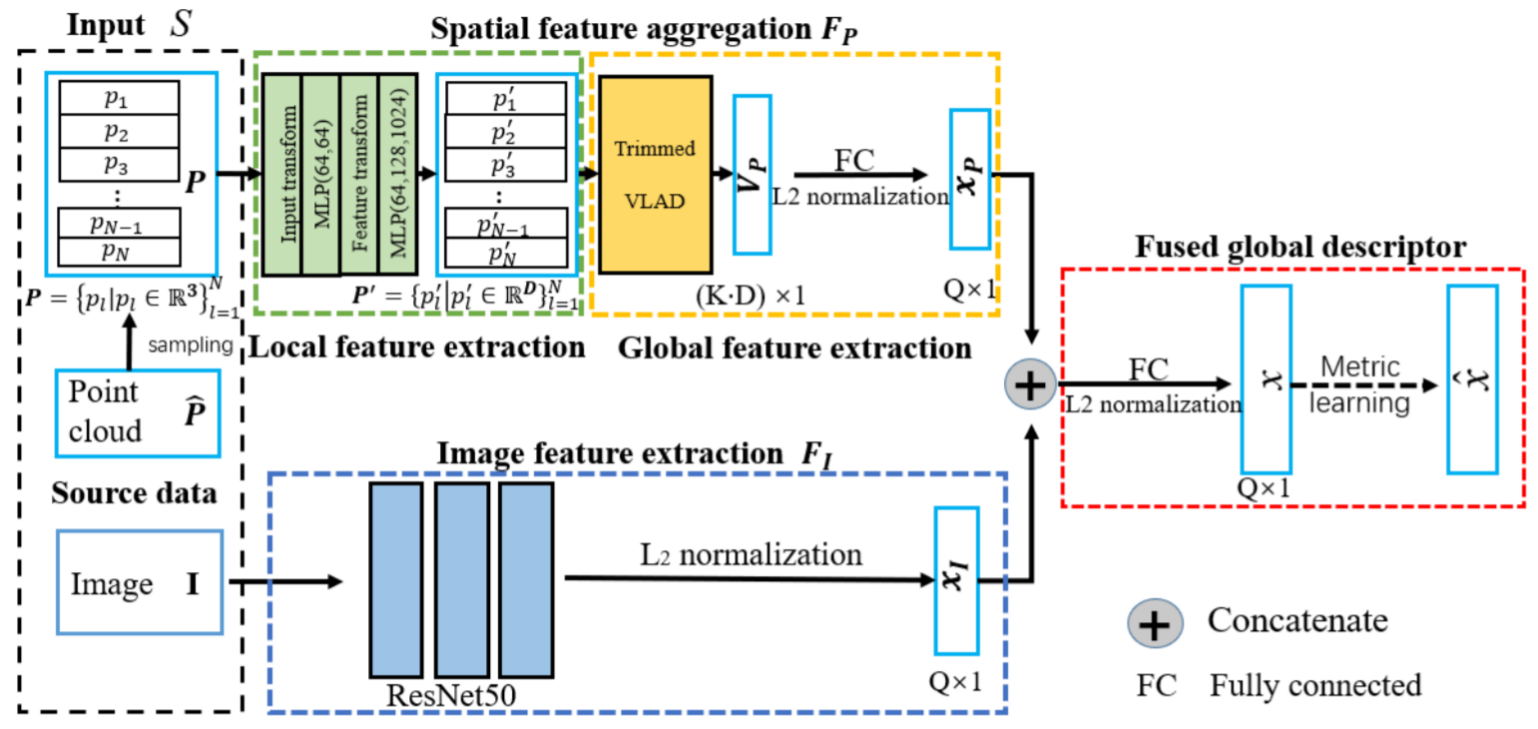
CNN based Camera-LiDAR Fused descriptor [88] for place recognition.

The image information and corresponding 3D PC were used as source input data. The PC data acquired form the LiDAR may vary in sizes. Deep learning based down-sampling preprocess was applied to extract features from 3D source PC. It then used NN for generating compact representation of a place. CNN performed the place retrieval by learning mapping from the input data space S = (I, P) to a new space. The whole framework for place recognition in Figure 36 showed that mapping was performed by efficient feature extraction operator (blue, green and yellow blocks) and using the similarity metric for the evaluation feature descriptors (red block).

They applied MLP and feature transform for local spatial feature extraction by mapping each 3D dimensional point into higher dimensional space. The local rotation invariant spatial features extracted by the CNN are in green block. It also introduced novel trimmed VLAD block for PC in which redundant information and environment disturbance were avoided by ignoring non-informative 3D PC clusters. It assigned the trimmed weight to meaningful clusters in partial aggregation process for obtaining the global descriptor (yellow block). It applied intra-normalization before vector concatenation, followed by L2 norm.

After the trimmed VLAD block, it used the fully connected layer to obtain useful features for Q-dimension compact global descriptor. Images contain many appearance-based features, which have mutual effects on the PC features. Features of camera-based images were extracted using ResNet50 [108] while the additional LiDAR sensor data was used to improve the place recognition in fused network. As a result, ResNet50 was used as image feature extractor, followed by L2 norm to make image and PC components in equal weights.

**Figure 37:**
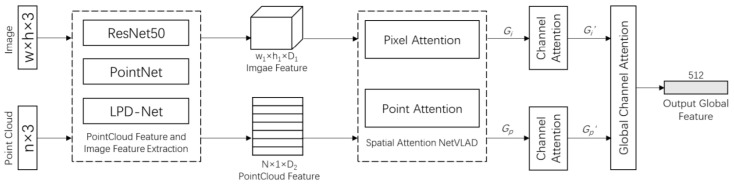
PIC-Net [89] composed of image and point cloud branch with spatial, channel, and global attention for large-scale place recognition.

Lu et al. [89] proposed a PC and image collaboration network (PIC-Net) shown in Figure 37 that fused image and PC features by attention method using DL approaches for large-scale place recognition. It mined the information of camera image with LiDAR PC and improved the place recognition performance by transforming the night image into daytime style. It used Resnet50 [108] to obtain a feature map from the image while PointNet [121] or LPD-Net [83] to extract features from PC. Then, both types of features were passed to the spatial attention layer for finding discriminative pixels and points with global channel attention layers for enhancing the features. Finally, the output of these three layers was used to generate final global features using an attention-based collaboration module.

The local spatial attention module shown was used in both images and point clouds for the selection of discriminative pixels and points. As shown in Figure 37, the PointNet and LPD-Net both were used for point feature extraction, while ResNet50 (after removing the final pooling layer) was used for image feature extraction. It aimed at learning the spatial attention map of the image and PC as well as adding the attention map to the feature aggregation. NetVLAD was used for aggregating the local features. It learned the cluster centers and calculated the residual, which was weighted by learnable parameter and attention weight of correspond pixel or point.

PCAN was replaced with a 1 × 1 × D1 convolution layer for point cloud and 1 × 1 × D2 convolution layer for image attention map learning. The Local channel attention module was used for learning the channel attention map to enhance the features from both PC and image branch before their fusion. For this, fully connected layer was implemented and then attention map was used to re-weight both image and PC features. The global channel attention was proposed to choose reliable features from PC and image branch.

For this, global channel attention map was learned using fully connected layer for the selection of reliable features.

##### Summary

We briefly summarize the 3DPR based on the reviewed methods in Section 3.2, which are listed in Table 19. Applications of 3DPR were vastly more researched in outdoor environments (10 vs. indoor environment two studies) based on sensor modalities that include camera-based (two studies), LiDAR-based (eight studies), and camera-LiDAR fusion (two studies). These studies show that current DL-based approaches use convolutional techniques for place recognition [139]. Convolutional place recognition approaches for indoor and outdoor environments are an extension of object recognition techniques. However, they are more concentrated on larger scale targets called the place landmarks [129].

Reliable place recognition is a challenging task due to changes in the environment and sensory ambiguity. Through the investigated studies, we found that LiDAR-based 3DPR methods were more robust to illumination, viewpoint change, and seasonal variations, which makes them competitive for outdoor 3DPR because of their longer-range capability compared to RGB-D cameras. Recent research work is more focused on DL-based applications in ADV, which shows that the integration of sensor-fusion process with recognition-based network structure for 3DPR is difficult.

However, studies show that the 3DPR task can be improved by considering the idea of using one sensor data to supervise the data of other sensors and integrating the map with sensor data for providing better environmental information to improve the detection. Furthermore, there is no optimal solution to handle the un-synchronization issue of multiple sensors. However, through the investigated studies, we found that its implicit solution can be learning from large-scale training data for landmark detection.

## 4. Datasets

Many public and new datasets have been developed for training the DL-based models. This section presents 3D datasets used in the studies that were reviewed in Section 3.1 and Section 3.2 for 3D object and place recognition tasks in the current review. We list the datasets used by each study in Table 20.

Several methods discussed in the survey illustrate that KITTI dataset [172] published in 2012 by [173] is the most frequently used dataset for 3DOR tasks. The review shows that many 3DOR models (19 out of 23 studies) have used the KITTI dataset. This dataset has been updated many times since its first release.

Current review shows that OXford RobotCar dataset [174] published in 2017 by [175] has gained attention from several ADV studies to perform 3DPR tasks. In the current survey, 7 out of 12 3DPR studies have used the Oxford RobotCar dataset. It contains over 1000 km of recorded driving of a consistent route with over 100 repetitions. It collected almost 20 million images from six cameras, along with the LiDAR and GPS.

A series of recent studies has also indicated that many research institutes have designed their datasets, such as the Waymo open dataset, HKUST, KAIST, and NYUD2 datasets.

Waymo is an open dataset [176] released recently by [177] for autonomous driving vehicles. It is a large dataset consisting of 1150 scenes and each scene is spanned 20 s. It is also well-synchronized dataset with 3D BBox in LiDAR data and 2D BBox in camera images. In this review, one study [62] used the Waymo dataset for training one-stage detector to recognize the objects in outdoor environment.

The HKUST dataset was captured by [82] for 3DPR task in their study. In this dataset, each shot is contained on a grayscale image and a point cloud. The KAIST dataset [178] was proposed by [179] to provide LiDAR and stereo images of complex urban scenes. One [88] among the reviewed studies used the KAIST dataset to perform 3DPR tasks. NYUD2 is a kinect dataset [180] that was used by one 3DPR study [78] in this survey. It was introduced by [181] with 1449 RGBD images and 26 scene classes of commercial and residential buildings.

Some networks (three 3DPR studies [80,83,86]) have used the in-house dataset that includes university sector, residential area, and business direct. This dataset was created by [80] using LiDAR sensors on the car driven in four regions at 10, 10, 8, and 5 km routes.

The SUN RGB-D dataset [182] used by one 3DPR [78] and two 3DOR [70,71] studies was presented by [183]. It contains 10,355 RGB-D scene images as training set and 2860 images as testing set for 3D object detection, which is fundamental for scene understating. ISIA RGB-D dataset is proposed by [78] for use in their own study for 3DPR task. It is a video dataset to evaluate RGB scene recognition videos. It contains more than five hours of footage of the indoor environment in 278 videos. it reuses 58 categories of the MIT indoor scene database [184].

The multi vehicle stereo event camera dataset also called MVSEC [185] is a collection of 3D perception data that was presented by [186] for event-based cameras. It has been used by the model in [79] to perform 3D place recognition task. Its stereo event data has been collected from a car, bike, handheld, and hexacopter in both indoor and outdoor environments.

The DDD17 dataset DDD17Dataset used in one 3DPR study [79] was introduced by [187]. It contains annotated dynamic and active-pixel vision sensors’ recordings, which consist of over 12 h of video in city driving at night, daytime, and evening in different weather conditions and vehicle speed. The ScanNet dataset was reported in [188]. It has been used by two 3DOR [70,71] and one 3DPR [81] studies in the current survey. It is an RGB-D video dataset containing 1513 scenes that are annotated with 3D camera poses. The research community has used this dataset for 3D scene understanding and semantic voxel labeling tasks.

The NCLT dataset [189] used by one 3DPR study [84] in this review, was documented in [190]. It is a long-term autonomy dataset for robotic research, which was collected using a Segway robot by 3D LiDAR, GPS, planar LiDAR along with proprioceptive sensors. Argoverse dataset [191] is introduced by [192] to support machine learning tasks for object detection in outdoor environment. A recent study [65] in the survey used this dataset for 3DOR task. It is mainly designed for 3D tracking and motion forecasting. Its 3D tracking dataset contains 360° images taken from seven cameras with 3D point clouds from LiDAR while its motion forecasting dataset contains 300,000 tracked scenarios. It also includes 290 km “HD maps”.

### Summary

Section 4 presented 14 datasets that have been used by 35 studies. The Sun RGB-D, KITTI, and ScanNet datasets have been used for both 3DOR and 3DPR tasks. However, KITTI is the most frequently used dataset for 3DOR tasks (used by 20/23 studies), while Oxford Robot-car is a widely used dataset for scene understanding to perform 3DPR tasks (7/12 studies) in autonomous driving vehicles.

## 5. Performance Evaluation

Section 5 analyzes and compares the existing results in the context of different datasets (discussed in Section 4) to present the performance of the methods that have reviewed in Section 3.1 and Section 3.2 for 3DOR and 3DPR tasks. The evaluation metrics that have been used for the KITTI dataset include average precision (AP) of Intersection over Union (IoU) for both bird’s eye view (AP${}_{bev}$) and 3D object detection (AP${}_{3D}$) along with the average orientation similarity (AOS) [173] and average localization precision (ALP). AP, AOS, and ALP metrics are divided into easy, moderate, and hard according to difficulty levels of 3D object detection, which are height, occlusion, and truncation for all three categories: cars, pedestrians, and cyclists. The recall @ 1 %, AUC, and accuracy % are the metrics that were used to compare the performance of 3DPR tasks on different 3D detection datasets.

For performance evaluation based on the KITTI dataset, Mono Pair [55] uses 40-point interpolated average precision metric AP${}_{40}$, which is evaluated at both the bird-eye view AP${}_{bev}$ and the 3D bounding box AP${}_{3d}$. It reports AP with intersection over union (IoU) using 0.7 as thresholds for cars, pedestrians and cyclists detection. Table 21 and Table 22 shows the performance of one-stage anchor-free detector of [55] on the KITTI validation and test sets for the car category, while performance for pedestrians and cyclists on the KITTI test is shown in Table 23 and Table 24, respectively. It can also perform inference in real-time as 57 ms per image, which is higher than [106].

GS3D [56] evaluated the framework on the KITTI object detection benchmark and follows [193] to use two train/validation (val) splits. Its experiments were mainly focused on the car category. Table 21 and Table 22 show the evaluation results of 3D detection accuracy on the KITTI for car category using the metric of AP${}_{3D}$ on two validation sets val${}_{1}$ and val${}_{2}$. The performance on val${}_{2}$ is higher than [102] for 3D object detection in autonomous driving. In [56], researchers used the metric of Average Localization Precision (ALP) and outperformed [193]. Table 21 presents the results of [56] for car category evaluated using the metric of ALP with the results on the two validation sets val${}_{1}$/val${}_{2}$.

SS3D [57] evaluated its proposed methods primarily on the KITTI object detection benchmark. It focused on three categories car, pedestrian and cyclist, which are most relevant for autonomous vehicle applications. The metric used for [57] evaluation is the average precision (AP), where valid detection is specified if the IoU is at 0.7, in bird’s-eye-view and in 3D, respectively.

The researchers in [57] used the same validation splits and called them split-1 [194] and split-2 [195], which divided the training data almost in half and performed the training on all three categories simultaneously. Table 21 shows AP with the 3D IoU detection criterion on validation set for the Cars class with a clear ranking Method 1 ≺ Method 2 ≺ Method 3 in terms of their performance. It also represents the results using the ALP metric. Jörgensen et al. [57] used inference on the KITTI test set and the evaluation results on test data for cars in Table 22, while pedestrians and cyclists classes in bird’s-eye-view (AP${}_{bv}$) and in 3D (AP${}_{3D}$) are presented in Table 24.

M3DSSD [58] evaluated the proposed framework on the challenging KITTI benchmark for 3D object detection covering three main categories of objects: cars, pedestrians, and cyclists. AP scores on validation and test sets of 3D object detection and bird’s eye view for cars are shown in Table 21 and Table 22, while the 3D detection performance for pedestrians and cyclists on test set at a 0.5 IoU threshold is reported in Table 24.

SRCNN [59] evaluated the proposed model using Average Precision for bird’s eye view (AP${}_{bv}$) and 3D box (AP${}_{3D}$) on the KITTI car validation and test sets, while the results are reported in Table 21 and Table 22, respectively. It outperforms state-of-the-art monocular-based methods [34,196] and stereo-method [197] by large margins. Specifically, for easy and moderate sets, it outperforms 3DOP [197] over 30% for both AP${}_{bev}$ and AP${}_{3D}$ while for the hard set, it achieved ∼25% improvements.

CenterNet [60] used restnet18 [108] and dla-34 [105] as backbone of its three methods and showed that its methods are superior to the previous monocular-based methods. The performance on AP${}_{bev}$ and AP${}_{3D}$ for car 3D localization and detection on the KITTI validation set is shown in Table 21.

RT3D [61] evaluated the proposed method on the KITTI for autonomous driving and divides the samples in training and validation sets exactly the same as [194]. The results of both 3D localization and 3D detection evaluations are obtained using Average Precision (AP${}_{loc}$) and (AP${}_{3D}$), as reported in Table 21 and Table 22 respectively. It is 2.5× faster than the [114]. Its detection time of 0.089 s allows it to be deployed in real-time systems and it achieves at least 13% higher accuracy compared to [102,194,198].

AFDet [62] evaluated the results using average precision (AP) metric as shown in Table 21, where the IoU threshold was 0.7 for the car class. They did not use complex post-processing process and NMS to filter out the results.

SegV Net [63] evaluated the 3D vehicle detection results on the KITTI test dataset using AP${}_{bev}$ and AP${}_{3D}$ metrics, as shown in Table 22, while the results on validation dataset with AP${}_{3D}$ metric and orientation estimation (AOS) are reported in Table 21. It outperformed LiDAR only single stage methods [111,113] in 3D vehicle detection.

SECONDX [64] supports cars, pedestrians and cyclists’ categories with a single model and outperforms other methods for all APs in three classes. Its evaluation results on the KITTI validation set are given in Table 21 and Table 23. It runs in real time without increasing memory usage and inference time compared with [120].

IPOD [66] follows AP metrics for all three classes where the IoU threshold is 0.7 for car class and 0.5 for pedestrians and cyclists classes. For evaluation on the test set, the model used train/val sets at a ratio of 4:1. The performance of the method is listed in Table 21, Table 22, Table 23 and Table 24. Yang et al. [66] showed that compared to [199], the detection accuracy of IPOD on hard set has improved by 2.52%, and 4.14% on BEV and 3D respectively. Similarly, compared to [73,120] it performs better in pedestrian prediction by 6.12%, 1.87%, and 1.51% on the easy, moderate, and hard levels, respectively.

FVNet [67] presents the performance for cars category at 0.7 IoU using AP${}_{bev}$ and AP${}_{3D}$ and for the pedestrians and cyclists categories at 0.5 IoU using AP${}_{3D}$ metric on the KITTI test dataset, as shown in Table 22 and Table 24. It achieved significant better results despite using the raw point clouds, and its inference time was 12 ms. Compared to [73], it performs best on all three categories except the car detection in easy setting, which employs both front-view and bird’s-eye-view.

In DPointNet [68], the dataset includes three categories of car, pedestrian, and cyclist. However, it only evaluates the car class for its rich data. Table 21 and Table 22 show its performance on the KITTI validation and test sets respectively using the average precision (AP) of car class with a 0.7 IoU threshold. Li et al. [68] demonstrated that the effectiveness of proposed DPointNet on the KITTI validation set has increased from 0.4% to 0.6%, with only about 60% running time.

Point-GCNN [69] used the KITTI benchmark to evaluate the average precision (AP) of three types of objects: car, pedestrian and cyclist. Following [111,114,200], it handles scale differences by training one network for the car and another network for both the pedestrian and cyclist. The AP results of 3D and BEV object detection on the KITTI test set for all three categories are shown in Table 22 and Table 24. It achieved good results for car detection on easy and moderate levels, for cyclist detection on moderate and hard levels while it surpasses previous approaches by 3.45. The reason of low pedestrian detection compared to its car and cyclist classes is that vertices are not dense enough to obtain more accurate bboxes.

S-AT GCN [72] evaluated 3D detection results using the 3D and BEV average precession at 0.7 IoU threshold for the car class and 0.5 IoU threshold for the pedestrian and cyclist classes. The results on the KITTI validation data are reported in Table 21 and Table 23. Its method 1 indicates the results of self-attention (AT) without dimension reduction while method 2 represents the results of self-attention with dimension reduction (ATRD). Compared to method 1, the second method performrf better for car detection on all three difficulty levels, pedestrians at the hard difficulty level, and cyclists at moderate and hard difficulty levels. Wang et al. [72] described that adding feature enhancement layer with self-attention, can bring extra 1% and 2–3% improvement for its pedestrians and cyclists’ detection.

MV3D [73] followed [194] to split training set and validation set, each containing about half of the whole dataset. It only focused on car category and performed the evaluation on three difficulty regimes: easy, moderate, and hard. The results using AP${}_{3D}$ and AP${}_{loc}$ at IoU = 0.7 on validation set are shown in Table 21. Chen et al. [73] has showed that the proposed method [73] performed better than [41] by AP${}_{loc}$ under IoU threshold 0.7 and achieves ∼45% higher AP${}_{loc}$ across easy, moderate, and hard regimes. Similarly it obtained ∼30% higher AP${}_{3D}$ over [41] with criteria of IoU = 0.7, and reaches at 71.29% AP${}_{3D}$ on easy level.

BEVLFVC [74] evaluated the pedestrian detection results using 3D detection average precision AP${}_{3D}$ on the KITTI validation dataset, as shown in Table 23. Wang et al. described that its highest performance on validation set can be achieved by fusing [114,126] with the proposed sparse non-homogeneous pooling layer and one-stage detection network.

D3PD [75] trained the model using different hyper parameters and evaluated the validation split using AP${}_{3D}$ metric for pedestrian detection, as shown in Table 23. Roth et al. [75] illustrated that the highest performance can be obtained using concatenation feature combination in the detection network and showed that deep fusion scheme performs slightly better than early fusion scheme.

MVX-Net [76] splits the training set into train and validation sets and does not include the samples from same sequences in both sets [73]. It evaluated the 3D car detection performance using AP metric in 3D and bird’s eye view for validation and test sets as shown in Table 21 and Table 22. The experimental results show that [76] with point fusion significantly improves the score of mean average precision.

SharedNet [77] achieves competitive results compared with other state-of-the-art methods. The results in the KITTI validation and test dataset for three classes (cars, pedestrians, and cyclists) were evaluated on mean average precision metric. The results for car validation and test set are given in Table 21 and Table 22 respectively while for pedestrian and cyclist categories on validation set are listed in Table 23. Wen et al. [77] illustrates that the proposed model [77] competes with [199,201] in comprehensive performance. For the cyclist class, it outperforms the [201] while in the car class, it is 2× faster than [201].

SDes-Net [86] trains and tests different descriptor extraction models on real world data from the KITTI dataset. It evaluates their performance for 3DPR tasks to determine matching and non-matching pairs of segments, and to obtain the correct candidate matches. First, it compares the general accuracy of different descriptors using positive and negative pairs of segments from the test set. The experimental results show that Siamese network [167] achieves the best overall classification accuracy, which is about 80%, listed in Table 25.

The second comparison among descriptors was conducted to find the potential descriptor for generating candidate matches based on the closest neighbor in the euclidean descriptor-space. The experimental results demonstrates that the group-based classifier and feature extraction network that was trained using contrastive loss function [165] performed the best with around 50% positive matches, while the Siamese network [167] had only around 30% positive matches.

OREOS [84] demonstrates the place recognition performance on NCLT and KITTI datasets for an increasing number of nearest place candidates retrieved from the map. with recall in % that is 96.7 on the KITTI dataset and 98.2 on NCLT dataset as shown in Table 25.

CLFD-Net [88] uses KITTI and KAIST datasets for place recognition task. KITTI dataset supplies 11 scenes containing accurate odometry ground truth information. These scenes are used in experiments and referred as KITTI 00, · · ·, KITTI 10. It has potential to be applied in the field of autonomous driving or robotic systems with a recall @1%. The performance is 98.1 for KITTI 00 scene, which is 1.7% higher than [80], and 2.5% higher than [108]. The performance on KAIST3two scene is 95.2, which is 8.5% higher than [80], and 6.9% higher than [108]. The overall performance of model [88] on the KITTI dataset with average recall @ 1% is higher than KAIST dataset as shown in Table 25.

Table 26 illustrates the performance of proposed network in [65] for vehicle and pedestrian detection using the standard average precision for 3D detection (AP${}_{3D}$) and on the bird’s eye view (AP${}_{bv}$). The AP scores are measured at IoU = 0.7 threshold for car class, and IoU = 0.5 for pedestrian class with a reasonable inference speed (30FPS).

RGNet [70] and HGNet [71] used the ScanNet and Sun RGB-D datasets to perform 3DOR tasks while [81] used ScanNet dataset for 3DPR task. In [70], the network model performs better on 15/18 classes for 3D object (i.e., chair, table, bed etc.) detection task using ScanNet dataset and evaluates the performance using mean average precession, which is given in Table 27 as model accuracy is 48.5 in terms of mAP @ 0.25. Its 3D object detection in point cloud on Sun RGB-D dataset showed the overall performance is 59.2 on 6/10 object classes with mAP @ 0.25.

In [71], 3D object detection results with 61.3 % accuracy on the ScanNet dataset has been achieved with mAP @ 0.25 while 61.6 % on Sun RGB-D dataset for the ten most commonly used object categories ( such as bed, sofa, chair, table etc). The results are listed in Table 27.

RGBD-Net [78] evaluated the scene recognition results on NYUD2, SUN RGB-D and the ISIA RGB-D dataset for 3DPR task. It follows the split by [181] to recognize 27 indoor categories of NYUD2 dataset into 10 categories. Scene categories in the SUN RGB-D dataset are 40 and in the ISIA RGB-D video database are eight. It contains 60 % data of each category for training and 40 % for testing. Following [183], it uses ther mean class accuracy for the evaluation and comparisons of results, which are shown in Table 27.

ISR-Net [81] uses the ScanNet benchmark to present the scene classification results for place recognition (library, bedroom, kitchen, etc) and achieves an average recall of 0.70 as shown in Table 27. It performs better on 11/13 scenes and jumps to 70.0% recall compared to [202], which has an average recall of at most 49.8%.

In Pointnetvlad [80], the performance on average recall at 1% is evaluated using the Oxford dataset and three in-house datasets. It achieved reasonable results, which are 80.31, 72.63, 60.27, and 65.3 for the Oxford, U.S., R.A., and B.D. datasets, respectively, as shown in Table 28.

MinkLoc3D [87] evaluated the experimental results on the Oxford dataset and three in-house datasets that were acquired using LiDARs with different characteristics. The evaluation results of place recognition model on Oxford Robot-car dataset have achieved 97.9 average recall at 1 %, which is higher than [83]. When [87] model is evaluated on three in-house datasets, its performance compared to [83] is 1.0 and 0.6 p.p. lower for U.S. and B.D. sets that is 95.0 and 88.5 respectively while 0.7 p.p. higher for R.A. set. The results are listed in Table 28.

The experimental results of PIC-Net [89] show the performance of its optimal configuration is 98.23% on average with the recall @ 1, as shown in Table 28, which is about 0.52% better than the direct concatenation.

Lpd-net [83] evaluated the network model on the three In-House datasets and achieved 96.00, 90.46 and 89.14 average recall @ 1 % for U.S., R.A., and B.D. sets, shown in Table 28. It is trained only on the Oxford Robotcar dataset and directly test it on the In-House dataset.

SDM-Net [85] considers ten place recognition cases and uses area under the precision-recall curve (AUC) to evaluate the sequence pairs for representative cases. The results for all of them are reported in Table 28. It outperforms [152], in six out of ten cases.

In Event-VPR [79] the performance of proposed method is evaluated on MVSEC and Oxford RobotCar datasets, and the results are listed in Table 28. On the MVSEC dataset, two daytime and three nighttime sequences are trained together, and then each of them is tested separately. The recall @ 1 % of its model in night sequences has achieved 97.05% on average while almost the same at daytime sequences. On the Oxford RobotCar dataset, it shows the model performance for place recognition under various weather and seasons. It uses night sequences for training and performs testing on the day and night sequences. Its recall @ 1 % on Oxford Robot-car dataset is about 26.02% higher than [203] but about 7.86% lower than [152].

### Summary

Section 5 analyzes the performance of the 3DOR and 3DPR methods by comparing the published results based on three evaluation metrics (AP, AOS, and ALP) for 3DOR and three evaluation metrics (Recall, Accuracy, and AUC) for 3DPR tasks. It classified the results for comparison according to the datasets used by each method.

Performance comparison on the KITTI car validation and test sets is presented inTable 21 and Table 22 respectively. Analysis on the KITTI pedestrian and cyclist validation set is given in Table 23 and on the test set is given in Table 24.

Table 21 shows that the performance of [77] on easy while [72] on moderate and hard difficulty levels is better for AP${}_{bev}$ (IoU @ 0.7); [63] on easy while [68] on moderate and hard levels performs better than the other methods for AP${}_{3D}$ (IoU @ 0.7); [61] val1${}_{1}$ set surpasses all models for ALP on all three levels.

Table 22 presents that [77] outperforms on easy while [69] performs better on moderate and hard sets for AP${}_{bev}$ (IoU @ 0.7); [69] performance is higher on all three levels compare to other methods for AP${}_{3D}$ (IoU @ 0.7); [63] model exceeds over [67] for AOS on all three levels.

In Table 23, the performance analysis of pedestrian category illustrates that [66] on all three levels outperforms for AP${}_{bev}$ (IoU @ 0.5); [77] on easy and moderate while [66] on hard level performs better for AP${}_{3D}$ (IoU @ 0.5). The comparison on cyclists category shows that first method of [72] on easy while its second method on moderate and hard levels gives better results using AP${}_{bev}$ (IoU @ 0.5); first method of [72] on moderate while its second method on easy and hard levels outperforms for for AP${}_{3D}$ (IoU @ 0.5).

Table 24 presents that, for the pedestrian category, the results of [55,66] outperform other methods on all three levels for AP${}_{bev}$ and AP${}_{3D}$ (IoU @ 0.7) and for AP${}_{bev}$ and AP${}_{3D}$ (IoU @ 0.5) respectively. For cyclist category the results of [69] and third method of [57] have higher performance on all three levels when compared using AP${}_{bev}$ and AP${}_{3D}$ (IoU @ 0.5) and AP${}_{bev}$ and AP${}_{3D}$ (IoU @ 0.7).

For 3DPR tasks, Table 25 presents that [88] has higher recall than [84] on the KITTI dataset while Constructrive and group-based methods have equally higher accuracy in [88]. Performance comparison for 3DOR task on ScanNet and Sun RGB-D datasets shows that [71] has higher mAP @0.25 compared to [70] in Table 27. Table 28 presents that [89] on Oxford Robot-car and [83] on the In-House datasets outperform [80,89] when evaluated with average recall @ 1 % for 3DPR task.

## 6. Discussion and Future Research Directions

This section summarizes the most relevant findings on the review of social representative robots (Section 2), camera and LiDAR-based data representation of 3D recognition (Section 3) for both object (Section 3.1) and place (Section 3.2).

This article first highlighted the value-centric role of social robots in the society by presenting recently developed robots. These social robots are performing front-line tasks and taking complex roles in public, domestic, hospitals, and industrial settings. The semantic understanding of the environment varies depending on the domain and application scenarios of the robots. For instance, the semantic understanding task for a robot working in a factory with a human co-worker is different from those robots working at home due to different objectives. Usually, these robots are equipped with a variety of sensors, such as camera and LiDAR to perform human-like recognition tasks.

Focusing on the recognition capability of social robots, it has explored camera and LiDAR-based 3D data representation methods using deep learning models for object and place recognition. Both sensors are affected by the changes in the scene lighting conditions as well as the other weather factors [204]. In addition, both object and place recognition (OPR) tasks rely on different methods of semantic understanding, which help to detect small and occluded objects in cluttered environment or objects in occluded scenes.

Examining the existing literature on 3D recognition reveals that there are relatively fewer studies on 3D place recognition compared to 3D object recognition. Moreover, a stable model for 3D recognition has not yet been formed. In the real world, a robot’s behavior strongly depends on its surrounding conditions and it needs to recognize its environment through the input scenery. However, literature search shows that up to now, little attention has been paid to LiDAR-based 3D recognition in indoor environment using DL-based approaches in contrast to outdoor recognition.

A monocular camera is a low-cost alternative for 3DOR and depth information is calculated with the aid of semantic properties understanding from segmentation. 3D monocular object detection can be improved by establishing pairwise spatial relationships or regressing 3D representation for 3D boxes in the indoor environment, while visual features of visible surfaces for extracting 3D structural information in the outdoor environment. Compared with the monocular camera more, precise depth information can be obtained through the stereo camera by utilizing semantic and geometric information and region-based alignment methods can be used for 3D object localization. However, it can be extended to general object detection by learning 3D object shapes.

At present, most of the 3DOR methods heavily depend on LiDAR data for accurate and precise depth information. However, LiDAR is expensive, and its perception range is relatively short. The article categorized the LiDAR-based 3DOR methods into structured, unstructured, and graph-based representations. Some 2D image grid-based methods used pre-RoI pooling convolution methods and pose-sensitive feature maps for accurate orientation and size that can be enhanced with a more advanced encoding scheme for maintaining height information.

We reviewed 3D voxel grid-based methods that incorporate semantic information by exploiting BEV semantic masks and depth aware head and by providing multi-class support for 3D recognition. 3D object detection from raw and sparse point cloud data has been far less explored to date using DL models, compared with its 2D counterpart.

3D LiDAR PC-based object detection can yield improved performance by context information and Precise PC coordinates as well as generating feature maps through cylindrical projection and combining proposal general and parameter estimation network. However, little research has looked into encoding PC using graph neural networks (GNNs) for highly accurate 3DOR. The joint learning of pseudo centers and direction vectors for utilizing multi-graphs was explored with supervised graph strategies for improving the performance. The point clouds do not well capture semantic (e.g., shape) information; however, utilizing the hierarchical graph network (HGNet) approach effectively handles this problem at multi-level semantics for 3DOR.

Sensor fusion methods based on camera and LiDAR for 3DOR using deep fusion schemes have gained attention. These methods rely on combing multi-view region-wise features, constructing sparse non-homogeneous pooling layer for feature transform between two views and allows fusion of these features, extracting point clouds using voxel feature encoder and utilizing anchor proposals, or integrating point and voxel fusions. In this direction, future research needs to deep multi-class detection network.

Unlike 3DOR, 3DPR task based on LiDAR and camera-LiDAR fusion methods by leveraging the recent success of deep networks has remained as a less explored problem. LiDAR PC based 3DPR methods depend on metric learning and inference to extract the global descriptors from 3D PC, extraction of local structures and finding the spatial distribution of local features, representation of semi-dense point clouds-based scene, utilization of data-driven descriptor for near-by place candidates, and estimation of yaw angle for oriented recognition. Camera-LiDAR sensors fusion methods to extract fused global descriptors for 3DPR via DL approaches depends on applying a trimmed strategy on the global feature aggregation of PC or using attention-based fusion methods to distinguish discriminative features that can be improved by color normalization.

## 7. Conclusions

To conclude, the present article began by enumerating the role of social robots as human assistants. Then, in the context of social robot capabilities, we focused on the recent publications related to the camera and LiDAR-based 3D data representation approaches for object and place recognition using the DL model between the years 2014 and 2021. This is the first combined study to review both 3D object and place recognition as well as recently developed social robots. We started by presenting the impact of social robots in the human-centric environment as a companion to tackle the daily problems in different (domestic, industrial, and medical) fields of life.

We described these recent robotic systems and listed their sensors, tasks, algorithms, appearances, semantic functions, and development status. Afterward, followed by the recognition capability of these social robots, we explored 3D data representation methods for object and place recognition based on camera and LiDAR using DL-based approaches with their advantages and limitations. In addition, we reviewed 3D detection datasets and present comparisons of the existing results.

To motivate those who are interested in DL-based 3D visual recognition approaches, the current study provides information in easy-to-understand tables, in particular, by pointing out the limitations and future research areas. In addition, this study describes different 3D datasets. Moreover, in this article, we analyzed and compared the existing results in the references for different datasets. Finally, we concluded the current survey with a discussion that suggests some promising research directions for future work.

## Figures and Tables

**Table 1:** The Contributions of This Survey.

Covered Topics	Guo et al. [43]	Sing et al. [44]	This Survey
Representative Social Robotic Systems	No	No	Yes [45,46,47,48,49,50,51,52,53,54]
3D Object Recognition (3DOR)	Yes	Yes	Yes [55,56,57,58,59,60,61,62,63,64,65,66,67,68,69,70,71,72,73,74,75,76,77]
3D Place Recognition (3DPR)	No	No	Yes [78,79,80,81,82,83,84,85,86,87,88,89]

**Table 2:** 3D Recognition: Inclusion and Exclusion Criteria.

	Inclusion Criteria	Exclusion Criteria
**Time Period**	2014–2021	Before the year 2014
**Domain**	Robotic System	Non-robotic system
**Articles’ Type**	Journal and conference publication	Text book chapters, encyclopedia, and posters
**Subject Area**	3D object and place recognition	2D object and place recognition
**Approaches**	Deep learning, neural networks, and CNN	Traditional methods
**Sensors**	Camera and LiDAR	Radar, GPS, and Ultrasonic

**Table 3:** Robots in Public Spaces.

	Amazon Scout [45]	AIM BOT [46]
**Sensor(s)**	Array of cameras and ultrasonic sensors	HD, infrared, RGB-D, surround and facial recognition cameras, ultrasonic and Pressure sensors, and high precision LiDAR
**Purpose/Usability**	Parcel delivery to destination.	Anti- epidemic COVID-19 protection assistant
**Scenario**	People monitoring scenario for epidemic prevention in indoor crowded places	Safe package delivery scenario in a robot carrier with potential benefits of immediate and cheap service
**Task**	People and pets’ recognition. Obstacle and sidewalks detection	Face recognition Mask detection
**Algorithm**	Machine Learning	3D Detection USLAM
**Appearance**	Wheeled robot	Autonomous driving mobile base robot
**Semantic Functions**	To navigate in sidewalks and climb up front porch for parcel delivery	To provide contact-less long-distance human body temperature measurement and screening
**Commercially Available**	Yes	Yes

**Table 4:** Robots in Domestic Environments.

	Chef Bot [47]	Astro [48]
**Sensor(s)**	Cameras, internal and external sensors	Cameras and the full range of audio-video sensors
**Purpose/Usability**	Chef assistant in the Kitchen	Family companion
**Scenario**	Collaborative cooking scenario with AI powered chef assistance in kitchen	Human-robot interaction scenario to perform day-to-day home tasks
**Task**	Kitchen utensils recognition, Speech recognition	Object detection, facial recognition, target tracking, and human pose estimation
**Algorithm**	AI and vision-based algorithm	Computer Vision, AI and SLAM
**Appearance**	Arm- shaped robot	Wheeled robot with a screen
**Semantic Functions**	To help in cooking the meals on voice commands To recognize and manipulate kitchen appliances	To bring coke To help with video calls and conferencing To play music To dance and entertain the children To care the elders To perform remote home monitoring
**Commercially Available**	No Release Date	Yes

**Table 5:** Robots in Hospitals.

	Moxi [49]	Reception- istBot [50]	CareBot [50]	MAiRA [51]
**Sensor(s)**	LiDAR sensor from Velodyne, camera from Intel, arm from Kinova, and a gripper	Camera, microphone, and speaker	Camera, microphone, speaker, and various health care device sensor	3D Vision sensors, voice recognition sensors, Smart touch sensitive 6-DOF sensor
**Purpose/Usability**	Hospital Robot Assistant	Patient Greeting	Clinical staff Assistance	Surgical Procedures
**Scenario**	Relieves the pressure from the hospital nursing staff in clinical setting scenario by retrieving and bringing supplies to hospital rooms or delivering the samples to the laboratories	Human–robot interaction scenario to perform hospital receptionist task	Patient care scenario in everyday nursing practices	Clinical care scenario to perform robot assisted procedure
**Task**	Detect and recognize objects and people	Face detection, speech Recognition	Face detection, speech recognition, server communication	Object and face recognition, voice recognition, gesture detection, pose estimation
**Algorithm**	Object and people recognition, human guided learning, object manipulation	OpenRTM [95], Yujin voice engine	OpenRTM [95], Yujin voice engine	Object recognition, voice recognition, human detection, gesture recognition
**Appearance**	Compliant arm, hand, mobile base	Wheeled base, arms, touch screen	Wheeled base, touch screen	Robotic Arm
**Semantic Functions**	To help clinical staff, i.e., nurses to complete their task, such as item collection	To communicate with patients, gather personal information from patients, assign them to CareBot	To acquire data about patients’ health condition, assist the nurses, measure the vital signs, i.e., pulse rate and report the results to medical staff	To assists medical staff in complex medical procedures
**Commercially Available**	Yes	No (field testing)	No (field testing)	Yes

**Table 6:** Robots in Industrial Environments.

	Handle [52]	LARA [53]	Stretch [54]
**Sensor(s)**	Cameras, 2D and 3D sensors	3D Vision sensor, torque sensor	2D camera, depth sensor
**Purpose/Usability**	Warehouse robotic assistant	Collaborative manipulation task	Warehouse robotic assistant
**Scenario**	Material handling scenario in warehouse	Human and industrial robot collaborative scenario to perform manipulation task	Box handling scenario in warehouse
**Task**	3D Box detection	Object recognition and grasping	Object detection and localization, smart grasping
**Algorithm**	Deep Learning-based vision	AI and Deep Learning	AI and Deep Learning
**Appearance**	Wheeled Robot with a manipulator arm	Wheeled base with robotic arm	Mobile base with wheels, robotic arm
**Semantic Functions**	To move boxes in the warehouse, unload trucks and build pallets	To perform industrial manipulation tasks with more speed and precision.	To perform warehouse operations (box shifting and platte building) more efficiently
**Commercially Available**	No	No	Expected in 2022

**Table 7:** Comparison of Camera and LiDAR Sensors.

Sensors	Advantages	Limitations
**Camera**	Color distribution, better sensing of objects, detailed information about objects by capturing their fine textures, low cost	Limited field of view, not accurate position estimation, affected by illumination condition, limited ability to detect the distance
**LiDAR**	Wide field of view, high angle and range resolutions, accurate position estimation, can be used at night	Unstructured point cloud data, insufferable in fog, snow, and rain, cannot capture fine textures of objects, expensive

**Table 8:** Methodology and Limitations: Camera-based 3DOR.

	Camera
**Methodology**	Uses RGB image for object detection and predicts 2D BBoxes, which are inferred to generate 3D BBoxes by re-projection or BBox regression, computationally less expensive compared to other methods
**Limitation(s)**	Input image does not have depth information, which causes low localization performance and inaccurate object size estimation
**Research Gap**	CNN architectures for estimating the depth information need to be investigated to improve the detection results

**Table 9:** Literature Analysis: Camera-based 3D Object Detection Methods.

Model	Detector Category	Environment	Camera	Scenario	Advantage(s)	Limitation(s)
Mono Pair [55]	One-stage	Indoor	Monocular	Partially occluded objects scenario in case of autonomous driving systems	Refines 3D object detection based on spatial relationship Uses 3D distances of adjacent neighbors to detect partially occluded objects	Detects cars only and ignores detection of other classes
GS3D [56]	Two-stage	Outdoor	Monocular	3D object detection based on a single RGB image in the scenario of autonomous driving	Overcomes the feature ambiguity issue by employing the features of visible surfaces to discover information of 3D structures	Restricts object detection on the boundary of the image
SS3D [57]	One-stage	Indoor	Monocular	3D object detection scenario from a single view in case of autonomous system.	Detects 3D objects and fits corresponding 3D BBoxes by a joint architecture Improves performance by modeling heteroscedastic uncertainty	The internal ranking is less pronounced
M3D SSD [58]	One-stage	Outdoor	Monocular	Objects’ mismatching and misalignment scenario in the anchor size and the anchor center	overcomes the size mismatching in receptive fields and anchors Reduces the center misalignment of object and anchor	Does not detect well for small objects at a distance greater than 60m
SRCNN [59]	Two-stage	Outdoor	Stereo	Sparse, dense, semantic and geometric information retrieval scenario from stereo imagery	Uses sparse and dense, semantic, and geometric information for 3D object detection without acquiring depth input and 3D position supervision. Simultaneously detects and associates the objects for left and right images with small modifications	Doubled training set
Center Net [60]	Two-stage	Outdoor	Stereo	Stereo based 3D object detection scenario that does not require depth estimation and anchor boxes	Does not reply on anchor-based 2D detection methods Does not use depth estimation and LiDAR data Detects small target objects that are occluded	Anchor-free left and right association and back-end optimization require improvement

**Table 10:** Methodology and Limitation(s): 2D Image Grid-based 3DOR Methods.

	2D Image Grid
**Methodology**	Projects 3D point clouds into a 2D image grid, which is passed to CNN for object detection with 2D BBoxes The 3D BBoxes are inferred from 2D BBoxes by performing position and size regression
**Limitation(s)**	Projection of 3D point clouds onto a 2D image grid causes information loss, which leads to inaccurate spatial information compared to raw PC data
**Research Gap**	Encoding of the input image by hand-engineered features could be replaced with learned representations to improve detection results

**Table 11:** Literature Analysis: 2D Image Grid-based 3DOR Methods.

	RT3D [61]	AFDet [62]
**Detector Category**	Two-stage	One-stage
**Environment**	Outdoor	Outdoor
**Projection**	FV	BEV
**Scenario**	3D vehicle detection scenario for collision avoidance.	3D object detection scenario on embedding system that is anchor free and Non-Maximum Suppression free
**Advantage(s)**	Completes detection in a shorter time than the scan period of the LiDAR using pre-RoI pooling convolution and pose sensitive feature maps	Provides anchor-free and NMS-free end-to-end 3D object detection
**Limitation(s)**	Performance on the test dataset is not as good	Height information is not fully preserved

**Table 12:** Methodology and Limitation(s): 3D Voxel Grid-based 3DOR Methods.

	3D Voxel Grid
**Methodology**	Discretizes 3D point clouds into 3D voxel grid representation that preserves shape information and performs recognition using CNN or fully CNN
**Limitation(s)**	Empty cells in their sparse representation make it computationally inefficient, 3D convolutions result in increased inference time
**Research Gap**	Generating 3D region proposals could improve localization accuracy and reduce computational time

**Table 13:** Literature Analysis: 3D Voxel Grid-based 3D Object Recognition Methods.

	SegV Net [63]	SECONDX [64]	LidarMTL [65]
**Detector Category**	One-stage	Two-stage	Two-stage
**Environment**	Outdoor	Outdoor	Outdoor
**Projection**	BEV	FV	BEV
**Scenario**	Ambiguous vehicles identification scenario from point cloud	Multi class 3D object detection scenario with a single model	Dynamic object detection and static road understanding scenario
**Advantage(s)**	Encodes the semantic context information in the feature maps to distinguish ambiguous vehicle for better detection	Provides multiple class support in a single model.	Performs robust 3D object recognition in complicated environment Also useful for online localization
**Limitation(s)**	Partial occlusion leads to false positives	Performance is not satisfactory for all the classes (e.g., cyclist and pedestrian.	The necessity of using loss weights with grid search

**Table 14:** Literature Analysis: PointNet-based 3DOR Methods.

	IPOD [66]	FVNet [67]	DPointNet [68]
**Detector Category**	Two-stage	Two-stage	Two-stage
**Environment**	Outdoor	Outdoor	Outdoor
**Scenario**	Intensive point-based 3D object detection scenario	3D front view proposal generation scenario for extracting point-wise features from the extruded object points	Point-cloud-based 3D object detection scenario that involves density-oriented point net
**Advantage(s)**	Reduces redundancy and ambiguity by seeding each point with proposals, without losing localization information from PC data.	Provides multi-scale 3D object detection. Generates 3D proposals from the front view without using a camera	Does not require additional calculations for inference
**Limitation(s)**	Weak performance on cyclists’ class	Front view maps are not reliable for object detection in case of occlusion	Performance drops for “easy” instance due to mismatched test and validation data distribution

**Table 15:** Methodology and Limitation(s): PointNet-based 3DOR Methods.

	PointNet
**Methodology**	Raw 3D point clouds are directly passed to CNNs for class predictions and BBox estimations without converting 3D points to 2D-image and 3D-voxel grids
**Limitation(s)**	Processing the entire point cloud causes increased computational complexity Uses region proposals (RP) to restrict the number of points, however, generating RP on raw point clouds is difficult
**Research Gap**	Processing of whole point cloud and methods to limit the number of points needs to be further investigated

**Table 16:** Literature Analysis: Graph-based Representation for 3DOR.

	Point- GCNN [69]	RGNet [70]	HGNet [71]	S-AT GCN [72]
**Detector Category**	One-stage	Two-stage	Two-stage	Two-stage
**Environment**	Outdoor	Indoor	Indoor	Outdoor
**Scenario**	Object detection scenario from a LiDAR point cloud using Graph neural network	3D object proposal generation and relationship extraction scenario in point cloud using relation graph network	Raw point clouds processing scenario for direct 3D bounding box prediction.	Local geometrical feature extraction scenario
**Advantage(s)**	Detects multiple objects by predicting their category and shape in a single shot with auto registration mechanism	Extracts uniform appearance features by point attention pooling method Holds appearance and position relationship between 3D objects by building a relation graph	Learns semantics via hierarchical graph representation, Applies multi-level semantics by capturing the relationship of the points to detect 3D objects	FE layers boost the contrast ration of feature map and increase the 3D recognition (true positive) rate of the subsequent CNN for small and sparse objects
**Limitation(s)**	Does not maintain the accuracy with down sampled data for the hard and moderate levels	Gives poor performance for detecting thin objects	The ProRe module is not effective for object detection if object features had been adequately learned	Run-time speed drops with FE layers

**Table 17:** Methodology and Limitation(s): Camera-LiDAR fusion-based 3DOR Methods.

	Camera-LiDAR Fusion
**Methodology**	Uses multi-modal CNN to fuse both LiDAR 3D point cloud and camera images Shows state-of-the-art and robust detection performance by taking advantage of both sensors
**Limitation(s)**	Computationally expensive to use data from two different sensors Requires calibration between LiDAR and camera An appropriate representation of different sensor modalities is difficult and passing them to a fusion network is also challenging
**Research Gap**	More research should be focused on improving the fusion of different sensing modalities

**Table 18:** Literature Analysis: Camera-LiDAR Fusion-based 3D Object Recognition Methods.

Model	Detector Category	Environment	Scenario	Fusion Level	Advantage(s)	Limitation(s)
MV3D [73]	Two-stage	Outdoor	Multi-view feature fusion and 3D object proposal generation scenario	Early, Late, Deep	Introduces a deep fusion scheme for leveraging region-wise features from bird-eye and front view for multi-modalities’ interaction	The low LiDAR point density does not allow the detection of far objects that are captured by the camera The BEV-based region proposal network limits the recognition Detects cars only
BEVLFVC [74]	One-stage	Outdoor	Fusion scenario for LiDAR point cloud and camera-captured images in CNN	Middle	Exploits and fuses the whole feature map in contrast to previous fusion-based networks Generates high-quality proposal by fusion but boosts the speed by the fast one-stage fusion-based detector	Does not have superior LiDAR input representation Detects pedestrians only
D3PD [75]	Two-stage	Outdoor	3D person detection scenario in automotive scenes	Early, Late, Deep	Performs end-to-end learning on camera-LiDAR data and gives high-level sensor data representation	Dependent on ground plane estimation for finding 3D anchor proposals
MVX-Net [76]	One-stage.	Outdoor.	Integration scenario for RGB and point-cloud modalities.	Early, Middle.	Reduces false positives and negatives due to its effective multi-modal fusion.	Does not provide a multi-class detection network.
SharedNet [77]	One-stage.	Outdoor.	LiDAR-camera-based 3D object detection scenario with only one neural network for autonomous vehicles.	Early, Middle.	Achieving a good balance between accuracy and efficiency. Reduces the memory requirements and model training time.	Slightly inferior performance in case of car detection.

**Table 19:** Literature Analysis: 3D Place Recognition (3DPR) Methods.

Model	Environment	Scenario	Sensors	3D Place Recognition
RGBD-Net [78]	Indoor	Depth-specific features learning for scene recognition scenario	Camera	RGB-D:3D Depth Feature based
Event-VPR [79]	Outdoor	Event-based visual place recognition scenario in changing environment	Camera	Event-based
Pointnetvlad [80]	Outdoor	Point-cloud-based retrieval scenario for place recognition	LiDAR	Point Cloud based
ISR-Net [81]	Indoor	Indoor scene recognition scenario with 3D scene representations (point clouds or voxels)	LiDAR	Point Cloud based
PCPR-Net [82]	Outdoor	Point-cloud-based place recognition scenario using hierarchical features extraction with CNN	LiDAR	Point Cloud based
Lpd-net [83]	Outdoor	Large scale place recognition scenario with feature extraction using global descriptors	LiDAR	Point Cloud based
OREOS [84]	Outdoor	Oriented recognition scenario to retrieve nearby place candidates	LiDAR	Point Cloud based
SDM-Net [85]	Outdoor	Place recognition scenario from a scene’s structure with semi-dense point clouds	LiDAR	3D-voxel grid
SDes-Net [86]	Outdoor	3D segment based on learned descriptors for place recognition scenario	LiDAR.	Point Cloud based
MinkLoc3D [87]	Outdoor	Place recognition scenario with discriminative 3D point cloud descriptor.	LiDAR.	Sparse voxelized point-cloud-based
CLFD-Net [88]	Outdoor	Fused global feature generation scenario for place recognition scenario	Camera, LiDAR	Image and Point Cloud based Fusion
PIC-Net [89]	Outdoor	Fusion based Place recognition scenario based on image and point clouds	Camera, LiDAR	Image and Point Cloud based Fusion

**Table 20:** Literature Analysis: Datasets.

		Ref #	A	B	C	D	E	G	H	I	K	L	M	N	O	P
3DOR	Table 9	MonoPair [55]							o							
		GS3D [56]							o							
		SS3D [57]							o							
		M3DSSD [58]							o							
		SRCNN [59]							o							
		CenterNet [60]							o							
	Table 11	RT3D [61]							o							
		AFDet [62]							o						o	
	Table 13	SegV Net [63]							o							
		SECONDX [64]							o							
		LidarMTL [65]										o				
	Table 14	IPOD [66]							o							
		FVNet [67]							o							
		DPointNet [68]							o							
	Table 16	Point-GCNN [69]							o							
		RGNet [70]					o						o			
		HGNet [71]					o						o			
		S-AT GCN [72]							o							
	Table 18	MV3D [73]							o							
		BEVLFVC [74]							o							
		D3PD [75]							o							
		MVX-Net [76]							o							
		SharedNet [77]							o							
3DPR	Table 19	RGBD-Net [78]	o			o	o									
		Event-VPR [79]								o				o		o
		Pointnetvlad [80]						o		o						
		ISR-Net [81]											o			
		PCPR-Net [82]		o												
		Lpd-net [83]						o		o						
		OREOS [84]							o		o					
		SDM-Net [85]								o						
		SDes-Net [86]							o							
		MinkLoc3D [87]						o		o						
		CLFD-Net [88]			o				o							
		PIC-Net [89]								o						

A: ISIA RGB-D; B: HKUST; C: KAIST; D: NYUD2; E: Sun RGB-D; G: In-House; H: KITTI; I: Oxford Robot-car; K: NCLT; L: Argoverse; M: ScanNet; N: DDD17; O: Waymo; P: MVSEC.

**Table 21:** Comparison of the Results on the KITTI Validation Dataset for the Car Category.

KITTI Validation Dataset (Category: Car)									
Task: 3DOR									
	**AP_*BV*_ (IoU @ 0.7)**			**AP_3*D*_ (IoU @ 0.7)**			**ALP**		
**Ref**	**Easy**	**Moderate**	**Hard**	**Easy**	**Moderate**	**Hard**	**Easy**	**Moderate**	**Hard**
[55]	24.12	18.17	15.76	16.28	12.30	10.42	-	-	-
[56]	-	-	-	13.46/11.63	10.97/10.51	10.38/10.51	71.09/66.23	63.77/58.01	50.97/47.73
[57] (M1)	-	-	-	11.54/8.66	11.07/7.35	10.12/5.98	80.28/73.32	70.78/59.85	58.14/51.09
[57] (M2)	-	-	-	13.90/9.55	12.05/8.07	11.64/6.99	79.33/72.83	71.06/59.90	58.31/51.44
[57] (M3)	-	-	-	14.52/9.45	13.15/8.42	11.85/7.34	81.22/72.97	71.05/59.94	60.22/51.80
[58]	34.51	26.20	23.40	27.77	21.67	18.28	-	-	-
[59]	68.50	48.30	41.47	54.11	36.69	31.07	-	-	-
[60]	58.36	42.97	36.19	41.11	30.21	25.23	-	-	-
	65.31	50.49	44.1	51.13	38.87	33.47	-	-	-
	68.8	51.19	44.28	54.72	39.2	33.74	-	-	-
[61]	-	-	-	72.85	61.64	64.38	88.29/54.68	79.87/42.10	80.42/44.05
[62]	87.1	82.72	78.97	81.01	72.62	67.47	-	-	-
	88.91	84.69	79.83	85.18	75.33	69.18	-	-	-
	89.42	85.45	80.56	85.68	75.57	69.31	-	-	-
[63]	-	-	-	89.35	79.05	77.41	-	-	-
[64]	-	-	-	85.94	75.96	74.37	-	-	-
[66]	88.3	86.4	84.6	84.1	76.4	75.3	-	-	-
[68]	-	-	-	89.27	79.28	78.35	-	-	-
[72]	88.84	86.79	85.41	86.03	76.95	75.52	-	-	-
	89.7	87.63	86.07	86.54	77.5	76.16	-	-	-
[73]	-	-	-	71.29	62.68	56.56	86.55	78.1	76.67
[76]	89.5	84.9	79.0	85.5	73.3	67.4	-	-	-
[77]	89.75	86.97	85.42	88.04	77.60	76.23	-	-	-

**Table 22:** Comparison of the Results on the KITTI Test Dataset for the Car Category.

KITTI Test Dataset (Category: Car)									
Task: 3DOR									
	**AP_*BV*_ (IoU @ 0.7)**			**AP_3*D*_ (IoU @ 0.7)**			**AOS**		
**Ref**	**Easy**	**Moderate**	**Hard**	**Easy**	**Moderate**	**Hard**	**Easy**	**Moderate**	**Hard**
[55]	19.28	14.83	12.89	13.04	9.99	8.65	-	-	-
[56]	-	-	-	7.69	6.29	6.16	-	-	-
[57] (3)	11.74	9.58	7.77	11.74	9.58	7.77	-	-	-
[58]	24.15	15.93	12.11	17.51	11.46	8.98	-	-	-
[59]	61.67	43.87	36.44	49.23	34.05	28.39	-	-	-
[61]	-	-	-	23.49	21.27	19.81	-	-	-
[63]	88.62	86.16	78.68	84.19	75.81	67.80	90.5	88.88	87.34
[66]	86.93	83.98	77.85	79.75	72.57	66.33	-	-	-
[67]	78.04	65.03	57.89	65.43	57.34	51.85	85.94	76.84	68.9
[68]	-	-	-	81.67	76.34	70.34	-	-	-
[69]	93.11	89.17	83.9	88.33	79.47	72.29	-	-	-
[76]	89.2	85.9	78.1	83.2	72.7	65.2	-	-	-
[77]	89.61	85.08	80.42	81.11	72.93	67.24	-	-	-

**Table 23:** Comparison of the Results on the KITTI Validation Dataset for the Pedestrian and Cyclist Categories.

KITTI (Val Data)												
Task: 3DOR												
	Category: Pedistrian						Category: Cyclist					
	**AP_*BV*_ (IoU @ 0.7)**			**AP_3*D*_ (IoU @ 0.7)**			**AP_*BV*_ (IoU @ 0.7)**			**AP_3*D*_ (IoU @ 0.7)**		
**Ref**	**Easy**	**Moderate**	**Hard**	**Easy**	**Moderate**	**Hard**	**Easy**	**Moderate**	**Hard**	**Easy**	**Moderate**	**Hard**
[75]	-	-	-	53.47	47.06	41.49	-	-	-	-	-	-
	**AP_*BV*_ (IoU @ 0.5)**			**AP_3*D*_ (IoU @ 0.5)**			**AP_*BV*_ (IoU @ 0.5)**			**AP_3*D*_ (IoU @ 0.5)**		
**Ref**	**Easy**	**Moderate**	**Hard**	**Easy**	**Moderate**	**Hard**	**Easy**	**Moderate**	**Hard**	**Easy**	**Moderate**	**Hard**
[64]	-	-	-	57.07	53.1	47.19	-	-	-	78.85	60.71	58.93
[66]	72.4	67.8	59.7	69.6	62.3	54.6	84.3	61.8	57.7	81.9	57.1	54.6
[72] (1)	64.06	58.93	55.24	58.52	54.54	50.46	85.19	71.06	67.1	82.55	67.6	62.69
[72] (2)	63.52	58.51	55.38	58.62	54.16	50.02	84.77	71.8	68.25	82.97	66.39	63.61
[74]	51.3	45.0	40.02	-	-	-	-	-	-	-	-	-
[77]	71.67	64.22	61.03	66.65	60.49	54.51	81.03	63.5	61.06	75.87	60.07	55.87

**Table 24:** Comparison of the Results on the KITTI Test Dataset for the Pedestrian and Cyclist Categories.

KITTI (Test Data)												
Task: 3DOR												
	Category: Pedistrian						Category: Cyclist					
	**AP_*BV*_ (IoU @ 0.7)**			**AP_3*D*_ (IoU @ 0.7)**			**AP_*BV*_ (IoU @ 0.7)**			**AP_3*D*_ (IoU @ 0.7)**		
**Ref**	**Easy**	**Moderate**	**Hard**	**Easy**	**Moderate**	**Hard**	**Easy**	**Moderate**	**Hard**	**Easy**	**Moderate**	**Hard**
[55]	10.99	7.04	6.29	10.02	6.68	5.53	4.76	2.87	2.42	3.79	2.12	1.83
[57] (3)	3.86	3.52	2.5	3.52	3.28	2.37	11.52	9.65	9.09	10.84	9.09	9.09
	**AP_*BV*_ (IoU @ 0.5)**			**AP_3*D*_ (IoU @ 0.5)**			**AP_*BV*_ (IoU @ 0.5)**			**AP_3*D*_ (IoU @ 0.5)**		
**Ref**	**Easy**	**Moderate**	**Hard**	**Easy**	**Moderate**	**Hard**	**Easy**	**Moderate**	**Hard**	**Easy**	**Moderate**	**Hard**
[58]	6.2	4.66	3.99	5.16	3.87	3.08	2.7	2.01	1.75	2.1	1.75	1.58
[66]	60.83	51.24	45.4	56.92	44.68	42.39	77.1	58.92	51.01	71.4	53.46	48.34
[67]	-	-	-	42.01	34.02	28.43	-	-	-	38.03	24.58	22.1
[69]	55.36	47.07	44.61	51.92	43.77	40.14	81.17	67.28	59.67	78.6	63.48	57.08

**Table 25:** Comparison of the Results on the KITTI, NCLT, and KAIST Datasets.

KITTI Dataset						NCLT Dataset	KAIST Dataset
Task: 3DPR						Task: 3DPR	
	Accuracy			Ref	Recall @ 1 %	Recall @ 1 %	Recall @ 1 %
Ref	Descriptors	Pair of Matching Segments	Candidate Matching	[88]	98.1	-	95.2
[86]	Siamese	80%	30%	[84]	96.9	97.0	-
	Group-based	-	50%	-			
	Constructrive	-	50%				

**Table 26:** Comparison of the Results on the Argoverse Dataset.

Argoverse Dataset												
Task: 3DOR												
	Car						Pedestrian					
	**AP_*bev*_ (IoU @ 0.7)**			**AP_3*D*_ (IoU @ 0.7)**			**AP_*bev*_ (IoU @ 0.5)**			**AP_3*D*_ (IoU @ 0.5)**		
**Ref**	**Easy**	**Moderate**	**Hard**	**Easy**	**Moderate**	**Hard**	**Easy**	**Moderate**	**Hard**	**Easy**	**Moderate**	**Hard**
[65]	72.9	56.9	14.1	53.4	24.3	1.80	40.6	22.9	6.1	33.3	17.0	4.20

**Table 27:** Comparison of the Results on the ScanNet, Sun RGB-D, ISIA RGB-D, and NYUD2 Datasets.

	ScanNet Dataset	Sun RGB-D Dataset	ISIA RGB-D Dataset	NYUD2 Dataset
	**Task: 3DOR**			
**Ref**	**mAP @ 0.25**			
[70]	48.5	59.2	-	-
[71]	61.3	61.6	-	-
	**Task: 3DPR**			
	**Avg Recall**	**Accuracy** %		
[78]	-	53.8	58.3	67.5
[81]	0.70	-	-	-

**Table 28:** Comparison of the Results on the Oxford Robot-car, MVSEC, and In-House Datasets.

	Oxford Robot-Car											In-House		
												U.S.	R.A.	B.D.
	Task: 3DPR													
Ref	Recall @ 1 %													
[80]		80.09										72.63	60.27	65.3
[87]		97.9										95.0	91.2	88.5
[89]		98.23										-	-	-
[83]		-										96.0	90.46	89.14
		AUC												
[85]	Pair Sequence	1	2	3	4	5	6	7	8	9	10	-		
		0.774	0.736	0.583	0.419	0.764	0.557	0.489	0.599	0.443	0.594			
[79]		Oxford Robot-car and MVSEC										-		
		Recall @ 1 %												
		Day 1	Day 2	Night 1	Night 2	Night 3		Cloud	Rain	Snow	Night			
		99.51	91.52	98.67	95.11	97.37		91.81	90.95	93.29	91.80			

## Abbreviations

The list of acronyms and abbreviations used in this survey is given below:

DL	Deep Learning
CNN	Convolutional Neural Network
3DOR	3D Object Recognition
3DPR	3D Place Recognition
RoI	Region of Interest
R-CNN	Region-based Convolutional Neural Network
SSD	Single Shot MultiBox Detector
YOLO	You Only Look Once
PC	Point Cloud
AVOD	Aggregate View Object Detection
PG-Net	Proposal Generation network
PE-Net	Parameter Estimation network (PE-Net)
KITTI	Karlsruhe Institute of Technology and Toyota Technological Institute
HKUST	Hong Kong University of Science and Technology
KAIST	Korea Advanced Institute of Science and Technology
NYUD2	New York University Dataset version 2
NCLT	The University of Michigan North Campus Long-Term Vision
DDD17	DAVIS Driving Dataset 2017
sGD	Stochastic Gradient Descent
BBox	Bounding Box
ADV	Autonomous Driving Vehicle
GS3D	3D Guidance and using the Surface feature
SS3D	Single-Stage Monocular 3D
M3DSSD	Monocular 3D Single Stage object Detector
SRCNN	Stereo Recurrent Convolutional Neural Network
NMS	Non-Maximum Suppression
FV	Front-View
BEV	Bird’s-Eye View
SegVNet	Segmentation-based Voxel Network
VFE	Voxel Feature Encoder
LidarMTL	Lidar-based multi-task learning network
IPOD	Intensive Point-based Object Detector for Point Cloud
FVNet	Front-View proposal generation Network
DPointNet	Density-oriented Point Network
GCNN	Graph Convolutional Neural Network
RGNet	Relation Graph Network
HGNet	Hierarchical Graph Network
S-AT GCN	Spatial Attention Graph Convolution
MV3D	Multi-view 3D Network
MS-CNN	Multi Scale Convolutional Neural Network
S-AT	Spatial-Attention
BEVLFVC	Bird’s Eye View LIDAR point cloud and Front View Camera image
MVX-Net	Multimodal Voxelnet for 3d object detection
NetVLAD	Network for Vector of Locally Aggregated Descriptors
DGCNN	Dynamic graph Convolutional Neural Network
AUC	Area Under Curve
AP	Average Precision
LPD-Net	Large-scale Place Description
